# Structural basis of genomic RNA (gRNA) dimerization and packaging determinants of mouse mammary tumor virus (MMTV)

**DOI:** 10.1186/s12977-014-0096-6

**Published:** 2014-11-14

**Authors:** Suriya J Aktar, Valérie Vivet-Boudou, Lizna M Ali, Ayesha Jabeen, Rawan M Kalloush, Delphine Richer, Farah Mustafa, Roland Marquet, Tahir A Rizvi

**Affiliations:** Department of Microbiology & Immunology, College of Medicine and Health Sciences, United Arab Emirates University, P.O. Box 17666, Al Ain, United Arab Emirates; Architecture et Réactivité de l’ARN, CNRS, IBMC, Université de Strasbourg, 15 rue René Descartes, 67084 Strasbourg cedex, France; Department of Biochemistry, College of Medicine and Health Sciences, United Arab Emirates University, Al Ain, United Arab Emirates

**Keywords:** Mouse mammary tumor virus (MMTV), RNA dimerization, RNA packaging, palindromic (pal) sequence, Dimerization Initiation Site (DIS), RNA intermolecular interactions, RNA packaging signal, Long-range interaction (LRI)

## Abstract

**Background:**

One of the hallmarks of retroviral life cycle is the efficient and specific packaging of two copies of retroviral gRNA in the form of a non-covalent RNA dimer by the assembling virions. It is becoming increasingly clear that the process of dimerization is closely linked with gRNA packaging, and in some retroviruses, the latter depends on the former. Earlier mutational analysis of the 5’ end of the MMTV genome indicated that MMTV gRNA packaging determinants comprise sequences both within the 5’ untranslated region (5’ UTR) and the beginning of *gag*.

**Results:**

The RNA secondary structure of MMTV gRNA packaging sequences was elucidated employing selective 2’hydroxyl acylation analyzed by primer extension (SHAPE). SHAPE analyses revealed the presence of a U5/Gag long-range interaction (U5/Gag LRI), not predicted by minimum free-energy structure predictions that potentially stabilizes the global structure of this region. Structure conservation along with base-pair covariations between different strains of MMTV further supported the SHAPE-validated model. The 5’ region of the MMTV gRNA contains multiple palindromic (pal) sequences that could initiate intermolecular interaction during RNA dimerization. *In vitro* RNA dimerization, SHAPE analysis, and structure prediction approaches on a series of pal mutants revealed that MMTV RNA utilizes a palindromic point of contact to initiate intermolecular interactions between two gRNAs, leading to dimerization. This contact point resides within pal II (5’ CGGCCG 3’) at the 5’ UTR and contains a canonical “GC” dyad and therefore likely constitutes the MMTV RNA dimerization initiation site (DIS). Further analyses of these pal mutants employing *in vivo* genetic approaches indicate that pal II, as well as pal sequences located in the primer binding site (PBS) are both required for efficient MMTV gRNA packaging.

**Conclusions:**

Employing structural prediction, biochemical, and genetic approaches, we show that pal II functions as a primary point of contact between two MMTV RNAs, leading to gRNA dimerization and its subsequent encapsidation into the assembling virus particles. The results presented here enhance our understanding of the MMTV gRNA dimerization and packaging processes and the role of structural motifs with respect to RNA-RNA and possibly RNA-protein interactions that might be taking place during MMTV life cycle.

**Electronic supplementary material:**

The online version of this article (doi:10.1186/s12977-014-0096-6) contains supplementary material, which is available to authorized users.

## Background

An essential step in retroviral life cycle is the efficient and specific packaging of two copies of plus-strand full-length genomic RNA (gRNA) into the virus particle from a large pool of cellular and other viral RNAs in the cytoplasm (reviewed in [1-6]). Retroviral RNA packaging process involves the recognition of *cis-*acting packaging signal(s) by the zinc finger(s) in the nucleocapsid (NC) domain of the Gag polyprotein (reviewed in [1-4,7]). Over the years, it has been shown that the packaging signal(s) of different retroviruses reside at the 5’ end of the gRNA and include continuous or discontinuous sequences from R, U5, and 5′ untranslated (UTR) regions and extend into the 5’ end of the *gag* gene (reviewed in [1-3,8-10]).

The retroviral RNA genome is packaged as a non-covalent dimer and the processes of dimerization and packaging are closely interlinked (reviewed in [1-6,11]). The sequences responsible for retroviral gRNA packaging and dimerization of a variety of retroviruses map to the same ~100-400 nucleotides (nt) at the 5’ end of the gRNA, and in most cases are genetically indistinguishable (reviewed in [1-3]).

Recent retroviral RNA cross-packaging studies have shown that the specificity of retroviral gRNA packaging can be exchanged by substituting the packaging signals of genetically diverse retroviruses [12,13]. This has further been substantiated by the fact that heterodimers involving RNAs from two divergent retroviruses with no sequence homology can also be packaged (reviewed in [2,11,14]). In addition, a number of RNA cross- and co-packaging studies among diverse retroviruses suggest that the process of gRNA dimerization and packaging is likely to involve recognition of structural motifs rather than primary sequences [12,13,15-19]. Consistent with this, the packaging and dimerization sequences of almost all retroviruses have been shown to assume higher order structures comprising of various structural motifs that have been shown to mediate RNA-RNA and RNA-protein interactions during retroviral RNA dimerization and packaging (reviewed in [1-4,20].

Retroviral RNA dimerization is usually mediated by a palindromic (pal) sequence known as the dimerization initiation site (DIS) present in the 5’ region of the gRNA. In a number of retroviruses, the DIS has been shown to assume a hairpin structure containing a canonical “GC” dyad which interacts with the DIS loop on the second gRNA copy, resulting in a kissing loop interaction [21-26]; (further reviewed in [27]). Pal sequences involved in RNA dimerization have been identified in human and simian immunodeficiency viruses (HIV-1, HIV-2, and SIV) (reviewed in [5,6,25,28-32]), feline immunodeficiency virus (FIV) [23,33], Mason-Pfizer monkey virus (MPMV) [8,21], and avian leukosis virus (ALV) [34]. In addition to gRNA dimerization and packaging, mutations in the DIS have been shown to affect other steps in retroviral life cycle such as reverse transcription [25,29,32,35,36] and recombination [37-39]. Since the DIS has been shown to regulate the retroviral life cycle, it is therefore an attractive target for antiretroviral drugs, such as aminoglycosides [40,41].

Despite having been studied extensively, little is known about the molecular mechanisms of gRNA dimerization and packaging during the mouse mammary tumor virus (MMTV) life cycle [42,43]. An earlier study by Salmons et al., [44] suggested that MMTV harbors sequences responsible for gRNA packaging in the 5’ region of its genome. Employing a biologically relevant *in vivo* packaging and transduction assay for MMTV [45], we have recently identified a continuous region, spanning the beginning of R to 120 nt of *gag* that was found to be critical for MMTV gRNA packaging and propagation [10]. Folding algorithms predicted that these sequences fold into a higher order structure comprising of a number of stable structural motifs.

In this study, we employed the technique of selective 2’hydroxyl acylation analyzed by primer extension (SHAPE) [46-48] to validate and further refine the predicted higher order features of the MMTV packaging signal. The SHAPE-validated RNA secondary structure revealed four prominent pal sequences (pal I, pal II, primer binding site (PBS) pal, and pal III), and one or more of them could potentially be involved in initiating intermolecular interactions during MMTV gRNA dimerization. A systematic deletion/substitution analysis of different pal mutants using *in vitro* dimerization, *in vivo* packaging and transduction assays revealed that RNA dimerization, packaging and propagation processes of pal II mutants was greatly compromised. Results obtained from these complementary approaches indicate that pal II serves as the primary palindromic point of contact during intermolecular interactions, leading to the dimerization and RNA packaging processes during MMTV life cycle.

## Results

### Predicted secondary structure of the MMTV 5’ gRNA

**Figure 1:**
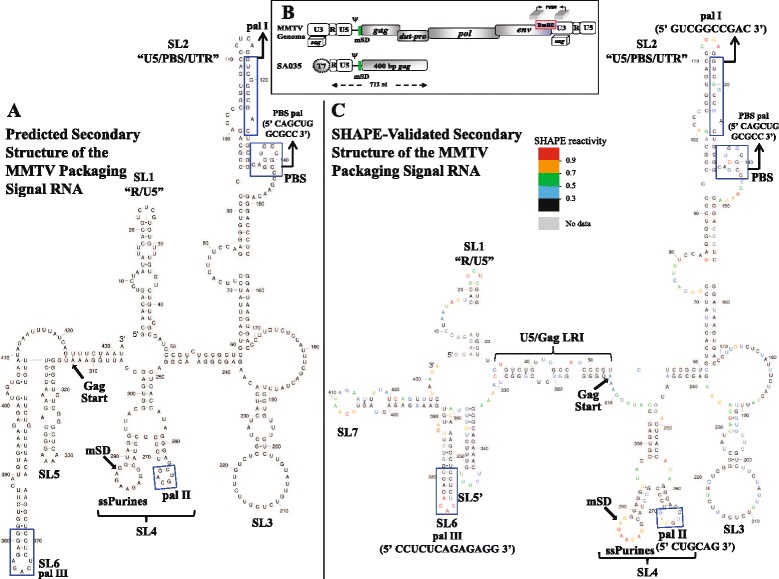
**Predicted and SHAPE-validated structural models of the MMTV packaging signal RNA. (A)** MMTV packaging signal RNA secondary structure predicted using Mfold. mSD, major splice donor, PBS, primer binding site. **(B)** Schematic representation of full length MMTV genome, showing the region used to create the T7 expression plasmid, SA035. **(C)** SHAPE-constrained RNA structure model of MMTV packaging signal. Nucleotides are color annotated as per the SHAPE reactivities key depending on their modification by BzCN. The data shown is an average of 3-6 independent experiments.

Using MMTV transfer vectors containing sequences from the 5’ end of the genome revealed that the sequences necessary for optimal MMTV gRNA packaging start at R and extend into first 120 nt of *gag* [10]. Therefore, the RNA secondary structure of the 5’ region of the MMTV gRNA containing 120 nt of *gag* (432 nt of the 5’ end from R) was predicted using Mfold. Specifically, structural analysis of this region predicted six stem loops 1-6 (SLs1-6, Figure 1A). Briefly, two stable stem loops, SL1 and SL2, were predicted in the R/U5/PBS/UTR region in addition to SL5 and SL6 in the *gag* region (Figure 1A). In between these stem loops, SL3 and a bifurcated SL4 were predicted in the UTR region. SL2 was found stably maintained in all predicted structures (data not shown). A closer look at the structure revealed the presence of four palindromic sequences, a 10 nt pal I in SL2, a pal sequence in PBS labelled as PBS pal, a 6 nt pal II in bifurcated SL4, and a 13 nt pal III in SL6 (Figure 1A). The PBS pal contained two overlapping pals (5’ *CAGCU****G*GCGCC** 3’; the first pal is in italics and the second pal is in bold letters (Figure 1A). Additionally, a 9 nt stretch of single-stranded purines (ssPurines) was also observed in SL4 which have been suggested to play a role in RNA-protein interaction during gRNA packaging [11,26]. The first apical loop of the bifurcated SL4 comprised pal II, while the second apical loop contained the ssPurines (Figure 1A).

### Validation of the secondary structure by SHAPE

To experimentally validate the predicted structure of the MMTV packaging signal RNA, we employed the SHAPE methodology [46-48]. Since ~50 nt at the 3’ end of the RNA molecule is lost due to primer binding for cDNA synthesis; therefore, to ensure that the entire 432 nt region of the 5’ end of the MMTV genome could be properly analyzed by SHAPE, we cloned a larger piece of RNA upto 712 nt in the wild type clone (SA035; Figure 1B) for *in vitro* transcription. Briefly, RNA transcribed from SA035 was purified, incubated in the dimer buffer, and modified with BzCN, as described earlier [21]. BzCN selectively acylates the 2’ hydroxyl group of ribose within nucleotides in flexible (unpaired) regions. Reactivity at each nucleotide was calculated by subtraction of the reverse transcription product of unmodified RNA from that of BzCN-modified RNA [21,49]. The SHAPE reactivity data obtained for each nucleotide from 3-6 independent experiments were then applied as pseudo-energy constraints in the structure prediction program “RNAstructure” in order to develop a refined RNA structural model of the sequences required for MMTV gRNA dimerization and packaging.

Consistent with the predicted structure, most of the loops and bulges containing unpaired nucleotides in the MMTV packaging signal RNA showed SHAPE reactivity (Figure 1C). We also observed moderate and high reactivities of nucleotides forming G-U base pairs, especially at the end of helices (G120, U320, G193, U294, G324, U356, U357, U358, U415, Figure 1C). Some base paired nucleotides (G21, C25, G84) located on either side of a bulge or loop also showed certain amount of reactivity, as expected. The refined structural model corroborated well with the predicted structure, supporting the existence of major structural motifs (SL1-SL6; Figure 1C). SHAPE also confirmed the existence of the bifurcated SL4 structure containing the 9 nt stretch of ssPurines and pal II, as predicted by Mfold (Figure 1C).

Even though a high conformity was observed between the predicted and the SHAPE-validated structural models, some conspicuous dissimilarity could be noted. In the SHAPE validated structure, SL1 and SL6 were shorter, resulting in the formation of SL7 which was not observed in the predicted structure (compare Figure 1A with Figure 1C). In addition, the sequences 5’ to the shortened SL6 form a new stem loop which is different from the predicted SL5 (Figure 1A) and therefore was labeled as SL5’ (Figure 1C). Interestingly, the single-stranded bulges and loops in and around SL3 did not show significant SHAPE reactivity despite being unpaired in the secondary structure model. This could possibly suggest that these nucleotides are involved in tertiary or inter/intramolecular interactions. Furthermore, the SHAPE-validated structure harbored a long-range interaction (LRI) involving complementary sequences from U5 and Gag (U5/Gag LRI) which was not predicted by Mfold (compare Figure 1A with Figure 1C). This U5/Gag LRI is made up of three short helices separated by a bulge and an internal loop (Figure 1C).

### The SHAPE-validated RNA structural model is supported by phylogeny

Sequence alignment of eight strains of MMTV (sequence from +1R-120 nt *gag*) revealed a high degree of conservation relative to the wild type *mtv*-1 (AF228550.1) strain, especially of sequences of the major structural motifs such as U5/Gag LRI, ssPurines, pal II, and PBS pal (Additional file 1A) once superimposed on the SHAPE-validated structure (Additional file 1B). Only a small number of nucleotides were observed to be variable (<75% conservation as shown in Additional file 1B). Finally, we generated a consensus structure of this region using RNAalifold (Additional file 2). Consistent with the SHAPE-validated structure (Figure 1C), consensus RNA secondary structure predictions (Additional file 2) revealed that the major structural motifs were consistently maintained. In addition, the consensus structures predicted by RNAalifold also supported the existence of SHAPE-validated short SL6 and SL5’ over the Mfold predicted long SL6 and SL5 motifs (compare Figure 1A, Figure 1C, and Additional file 2). The consensus structures also revealed the existence of LRI which is very similar to the one observed in the SHAPE-validated structure (Figure 1C and Additional file 2).

### Identification of the pal sequence mediating MMTV gRNA dimerization and packaging

The SHAPE-validated region found to be crucial for MMTV gRNA packaging revealed four pal sequences (Figure 1C) which could possibly play role(s) in intermolecular RNA interaction(s) to augment dimerization and subsequent packaging of the dimerized genome. Therefore, to ascertain which pal may mediate such interactions, we performed a systematic mutational analysis of these pal sequences and tested their effects on both RNA dimerization and packaging employing a combination of genetic and biochemical approaches.

#### Mutational analysis of pal I reveals that it does not play a role in MMTV gRNA dimerization and packaging

**Figure 2:**
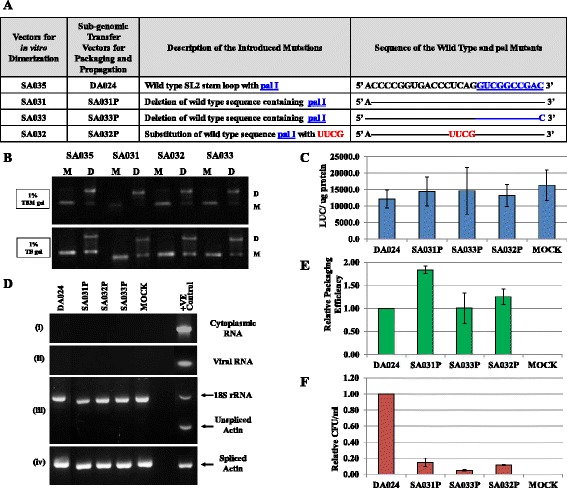
**Role of pal I in MMTV gRNA dimerization, packaging, and propagation. (A)** Description of the pal I mutants. The wild type pal I sequence is shown in blue and the mutations are depicted in red. **(B)** Typical RNA dimerization TBM and TB gels of wild type and mutant MMTV RNAs. M: monomer lane or monomer conformer; D: dimer lane or dimer conformer for each sample. **(C)** Transfection efficiencies of the mutants and wild type transfer vectors that were used to normalize the packaging efficiency. LUC: Luciferase activity. **(D)** PCR amplifications of the DNase treated cytoplasmic (panel i) and viral (panel ii) RNAs using virus specific primers. In the third panel (iii), amplification was conducted on the cDNAs obtained from cytoplasmic RNAs using primers that amplify unspliced β-actin mRNA. Multiplex amplifications were conducted in the presence of primers/competimer for 18S ribosomal RNA. The fourth panel (iv) shows PCR of cytoplasmic cDNA using primers that amplify spliced β-actin mRNA. **(E)** Relative packaging efficiency (RPE) of transfer vector RNAs. **(F)** Relative hygromycin resistance (Hyg^r^) colony forming unit per ml (CFU/ml) for mutant transfer vectors reflecting the relative RNA propagation efficiencies. In **(C)**, **(E)**, and **(F)**, the histograms represent data from at least three independent experiments (± SD). The *P* values for all mutants in panel **(F)** were significant (<0.001).

The mutations introduced in pal I are shown in Figure 2A. These mutations included a complete deletion of 26 nt of the apical stem loop of SL2 containing pal I, as well as simultaneous substitution of the deleted sequences with a UUCG stable tetraloop in order to maintain a stem loop structure without pal I [21]. It is important to mention that the attachment (*att*) sites needed for integration of the provirus were not affected in pal I mutants, thus allowing us to test the same mutations in both the *in vitro* dimerization as well as *in vivo* packaging and propagation assays [45]. As RNA dimers may have different stabilities, we analysed the RNA dimers by agarose gel electrophoresis in the presence of either TB or TBM buffers. The presence of Mg^2+^ ions minimizes dissociation of RNA dimers during electrophoresis, while weak dimers might dissociate during electrophoresis in TB buffer [34,50]. None of the deletion and/or deletion/substitution mutants showed any detrimental effects on dimerization when compared to the wild type clone SA035 (Figure 2B). The fold dimerization of the mutant clones (SA031-SA033) was observed to be between 0.88-1.04 in TBM gels and 1.29-1.82 in TB gels compared to the wild type, SA035 clone (data not shown). The increased amounts of dimers in the TB gels might reflect a stabilization of the mutant RNA dimeric species. Altogether, these data indicate that pal I does not play a major role in initiating intermolecular interactions between the two gRNAs.

To further evaluate the functional impact of mutations in pal I, we tested these mutants in a biologically-relevant *in vivo* packaging and propagation assay (Additional file 3). This was accomplished by cloning these mutations into the MMTV subgenomic transfer vector, DA024, creating SA031P-SA033P pal I mutant transfer vectors (Figure 2A). These transfer vectors were tested by transfecting human embryonic kidney (HEK) 293T cells along with the packaging and vesicular stomatitis virus glycoprotein (VSV-G) envelope expression constructs (Additional file 3). The wild type and mutant viral particles produced were used to: 1) measure the packaged viral RNA using qPCR, and 2) assess the propagation of the packaged RNA in the target cells which was monitored by the appearance of hygromycin resistant colonies. We first checked the transfection efficiency of the cultures containing mutant transfer vectors. The transfection efficiencies from at least three independent experiments were observed to be within 2 folds of each other, suggesting that all cultures were efficiently transfected (Figure 2C). These transfection efficiencies were taken into account to normalize and calculate the RNA packaging efficiency and propagation data for each mutant relative to the wild type.

To determine the RNA packaging efficiency, cDNAs were prepared from cytoplasmic and pelleted virion RNAs. Before making the cDNAs these RNAs were subjected to DNase treatment to ensure that there was no contaminating plasmid DNA, followed by PCR using virus-specific primers. Following amplification, the lack of a positive signal indicated that the DNA contamination in our RNA preparations was below the detection level (Figure 2D, panels i and ii). After having confirmed this, RNA preparations were reverse transcribed and cDNAs were prepared. Since the relative packaging efficiency data is expressed in relation to the efficiently and stably expressed viral RNAs that are exported from the nucleus to the cytoplasm, we monitored the integrity of the cytoplasmic RNA fraction by checking for the absence of unspliced β-actin mRNA by RT-PCR (Figure 2D, panel iii). To ensure that each cytoplasmic RNA fraction contained amplifiable cDNAs, the unspliced β-actin PCRs were conducted in the presence of primers/competimer for 18S ribosomal RNAs as an internal control [8,10,33]. In a separate amplification reaction, the same cytoplasmic cDNA samples were amplified using spliced β-actin primers (Figure 2D, panel iv). Figure 2D shows that while 18S ribosomal RNA and spliced β-actin mRNA were amplifiable, unspliced β-actin mRNA was not. These results indicated that the fractionation technique employed was robust and that the nuclear membrane integrity was maintained during the fractionation process.

In order to monitor the effect of pal I mutants on gRNA packaging, the relative expression of transfer vector RNAs in the cytoplasm was tested using a custom-made MMTV real time PCR assay in combination with a commercially available-endogenous β-actin Taqman assay as described earlier [10]. Following normalization of the RNA packaged into the virus particles to the cytoplasmic transfer vector RNA expression and to transfection efficiencies, the packaging efficiency of the transfer vector RNAs into the virus particles was measured (see Methods). The *in vivo* gRNA packaging data from multiple experiments revealed that none of the pal I mutations significantly decreased gRNA packaging (*P* >0.01; Figure 2E). This corroborated well with the dimerization data for the same mutants which showed no significant dimerization defect (Figure 2B). Interestingly, when viral particles containing RNAs from these mutants were used to transduce target cells with a *hygromycin resistance* gene, a drastic reduction of RNA propagation (6-20 fold reduction of the relative CFU/ml, *P* ≤0.001) was observed for all mutants (Figure 2F). Lack of RNA propagation of the packaged mutant transfer vector RNAs suggests that the introduced mutations impaired post packaging events of viral life cycle such as reverse transcription and/or integration since our readout assay is dependent on successful completion of these steps. Consistent with this hypothesis, mutations in close vicinity of the PBS (as has been the case for these pal I mutants) have been shown to greatly impinge reverse transcription [25,51,52].

#### Mutational analysis of pal II and the PBS pal suggests that there could be two points of contact between the gRNAs leading to MMTV gRNA dimerization

**Figure 3:**
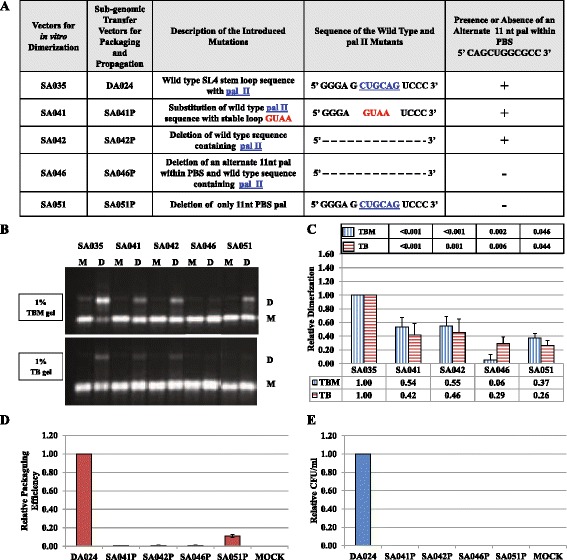
**Role of pal II and PBS pal in MMTV gRNA dimerization, RNA packaging and viral propagation. (A)** Description of the pal II mutants. **(B)** Typical RNA dimerization in TBM and TB gels of wild type and mutant MMTV RNAs. M: monomer lane or monomer conformer; D: dimer lane or dimer conformer. **(C)** Quantification of the relative RNA dimerization levels (see table below the histogram). Table above the histogram represent the *P* values of the respective mutants in TBM and TB gels. The experiments were repeated 2-6 times. **(D)** Relative packaging efficiency of transfer vector RNAs. **(E)** RNA propagation efficiency expressed as Hyg^r^ colony forming unit per ml (CFU/ml). In **(C)**, **(D)**, and **(E)**, the histograms represent data from at least three independent experiments (± SD). The *P* values for all mutants in panels **(D)** and **(E)** were significant (<0.001).

To test if pal II modulates MMTV gRNA dimerization and packaging, we introduced a series of similar mutations, including a complete deletion of pal II as well as substitution with a stable tetraloop sequence, in order to maintain the overall RNA secondary structure of this region (Figure 3A). Data obtained from 2-6 independent experiments revealed that a complete deletion of pal II resulted in a significant ~ two-fold decrease in RNA dimerization, both in TBM and TB gels (Figure 3B and Figure 3C, compare mutant SA042 with wild type SA035, *P* ≤ 0.001). Deletion of pal II with simultaneous substitution with a stable tetraloop had the same effect on RNA dimerization (Figure 3B and Figure 3C, mutant SA41; ~ two-fold reduction in relative RNA dimerization; *P* < 0.001).

**Figure 4:**
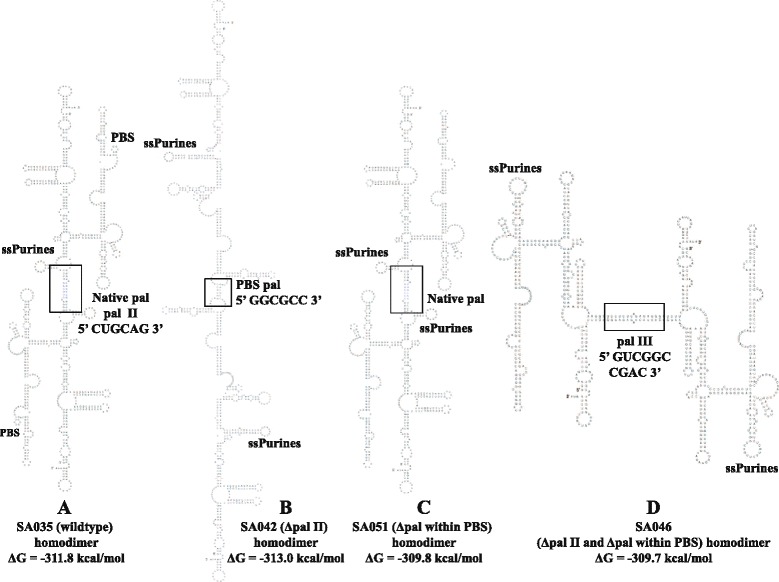
**Structural analysis of pal II and PBS pal deletion-mutant homodimers.** Predicted homodimer structures of **(A)** MMTV wild type (SA035), **(B)** pal II deletion (SA042), **(C)** deletion of the pal sequence within PBS (SA051) and **(D)** double deletion of both pal II and pal sequence within PBS (SA046). The dimers were predicted using RNAstructure and the contact point (point of dimerization) of the sequences between two RNAs leading to dimerization are boxed.

These results indicated that although pal II deletion mutants could reduce RNA dimerization, they could not completely abrogate it. Such an observation suggested the presence of another sequence that could facilitate dimerization in addition to pal II, though not to the wild type levels. Therefore, the pal II deletion mutant (SA042) sequence was folded as dimer using the RNAstructure software, which predicted that in the absence of pal II, the MMTV RNA packaging signal could potentially dimerize *via* the two overlapping pals in the PBS (5’ *CAGCU****G*GCGCC** 3’; the first pal is in italics and the second pal is in bold (Figure 4). To determine the role of PBS pal, if any, on dimerization, a mutant (SA051) was generated containing a complete deletion of the 11 nt overlapping pals within the PBS. Along the same lines, a double mutant (SA046) was created containing the deletion of pal II sequence as well as the overlapping pals within the PBS (Figure 3A). Deletion of the 11 nt PBS pal alone in SA051 resulted in 60% reduction in RNA dimerization levels (*P* ≤0.05; Figure 3B and Figure 3C). Deletion of both sequences (SA046) resulted in almost a complete abrogation of dimerization in TBM condition (18-fold; *P* <0.002) and to a 3.5-fold decrease in TB condition (*P* <0.006; Figure 3B and Figure 3C). These results suggest that the MMTV genome may require two points of contact during intermolecular interactions to augment efficient gRNA dimerization.

**Figure 5:**
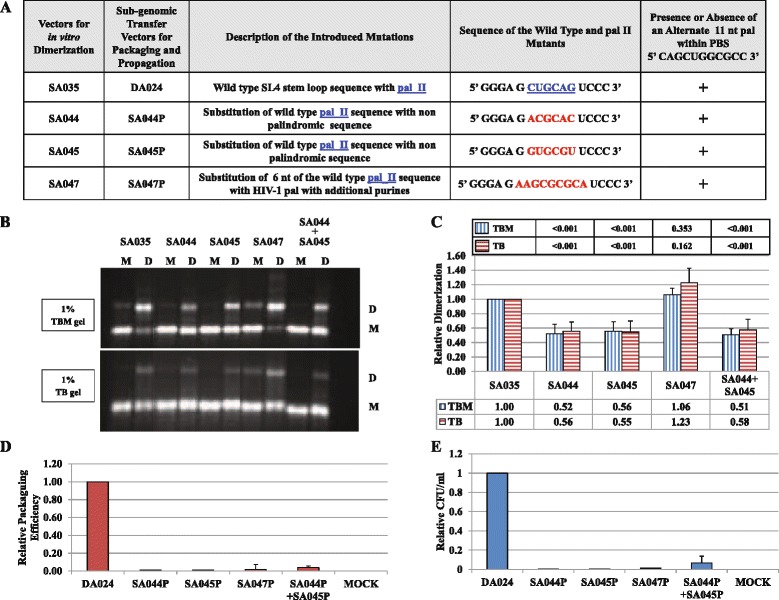
**Effects of pal II substitution mutations on MMTV gRNA dimerization, RNA packaging and viral propagation. (A)** Description of the pal II substitution mutants. **(B)** Typical RNA dimerization in TBM and TB gels of wild type and mutant MMTV RNAs. The last two lanes correspond to the co-incubation of *trans*-complementary mutants SA044 and SA045. M: monomer lane or monomer conformer; D: dimer lane or dimer conformer. **(C)** Quantification of the relative RNA dimerization (see table below the histogram). Table above the histogram represent the *P* values of the respective mutants in TBM and TB gels. The experiments were repeated 2-6 times. **(D)** Relative packaging efficiency of transfer vector RNAs. **(E)** RNA propagation efficiency expressed as relative Hyg^r^ colony forming unit per ml (CFU/ml). In **(C)**, **(D)**, and **(E)**, the histograms represent data from at least three independent experiments (± SD). The *P* values for all mutants in panels **(D)** and **(E)** were significant (<0.001).

Results in Figure 3 and Figure 4 showed that one of the points of contact between the two RNAs is pal II. To ascertain whether the primary sequence or the palindromic nature of pal II is important to initiate intermolecular interactions between the two RNAs during MMTV gRNA dimerization, we generated several additional mutants. The wild type pal II was substituted with HIV-1 pal flanked by additional purines (5’ AAGCGCGCA 3’; Figure 5A, mutant SA047). Two additional mutants were also created in which the pal II sequence was substituted by non-palindromic *trans-*complementary sequences (Figure 5A, mutants SA044 and SA045). In all of these mutants, the pal sequence within the PBS was maintained. Consistent with the effect of the purines flanking the pal sequence on HIV-1 RNA dimerization [26], replacing pal II by the HIV-1 pal and the flanking purines (SA047) preserved MMTV RNA dimerization to wild type levels (Figure 5B and Figure 5C). The *trans-*complementary mutants SA044 and SA045 RNAs were incubated either separately or together, allowing them to interact to initiate intermolecular interactions due to the *trans*-complementary nature of the sequences. When incubated separately, the mutants displayed a ~ two-fold decrease in dimerization (*P* <0.001; Figure 5B and Figure 5C), as expected similar to other pal II substitution and deletion mutants (Figures 3B and Figure 3C). Surprisingly, RNA dimerization was not restored when these *trans*-complementary mutants were co-incubated since the level of dimerization observed for the individual mutants was the same as when the equal amounts of two mutant RNAs were incubated together (two-fold decrease in dimerization; *P* <0.001; Figure 5B and Figure 5C). This is in sharp contrast with other retroviral systems in which dimerization has been restored to wild type levels in the mutants containing *trans-*complementary sequences [21,53-55].

### Role of pal II and PBS pal in regulating MMTV gRNA packaging and propagation

Next, we analyzed the RNA packaging and propagation efficiencies of the pal II and PBS pal mutants by cloning these mutations into the MMTV subgenomic transfer vector, DA024, similar for the pal I mutants, creating SA041P-SA047P and SA051P (Figure 3A and Figure 5A). Data from several experiments showed that pal II and PBS pal mutants’ transfection efficiencies were within two folds and that the transfer vector RNAs were expressed efficiently (data not shown). When similar amounts of virions were used to isolate packaged RNA, all of the mutants tested except for SA051P, were found to be severely impaired for gRNA packaging (27-150 fold reduction, *P* < 0.001, Figure 3D and Figure 5D). Although packaged, the relative packaging efficiency of SA051P mutant was reduced by 9-fold compared to the wild type (Figure 3D). Interestingly, deletion of either pal II or the PBS pal had a pronounced effect on RNA packaging (even though the effect of pal II deletion was more dramatic on packaging; Figure 3), suggesting that the MMTV Gag precursor initially recognizes an RNA dimer with two points of contact.

Propagation of these mutants, as measured by counting the number of hygromycin resistant colonies (CFU/ml) in the infected cultures correlated well with the significantly-reduced RNA packaging data (Figure 3E and Figure 5E). Lack of propagation of SA051P mutant can be explained despite residual RNA packaging by the deletion in the PBS, since our assay requires the packaged RNA to be successfully reverse transcribed (initiated by an intact PBS) before integration to allow expression of the *hygromycin resistant* gene and subsequent appearance of hygromycin-resistant colonies.

Altogether, the dimerization and packaging results suggest that the pal II and PBS pal play important roles in both MMTV gRNA dimerization and packaging. However, the case of mutant SA047P containing substitution of pal II with HIV-1 pal along with flanking purines was intriguing as its RNA packaging and propagation were abolished (Figure 5D and Figure 5E), despite displaying wild type RNA dimerization levels (Figure 5B and Figure 5C). These results suggest that RNA dimerization may not be sufficient to ensure RNA packaging and propagation and further indicate that pal II could also be involved in RNA-protein interactions during genome encapsidation.

### SHAPE analyses of pal II mutants support their role in MMTV gRNA dimerization

**Figure 6:**
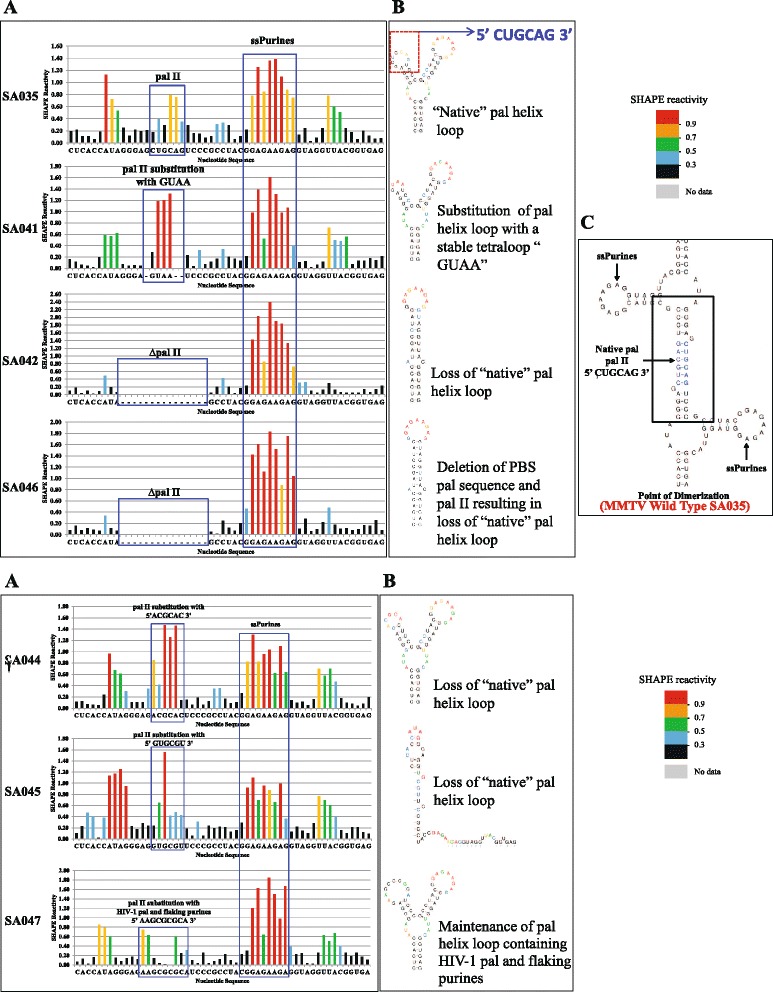
**SHAPE and structural analyses of the SL4 domain of the pal II and PBS pal mutants. (A)** SHAPE reactivity profile of the wild type and mutant RNAs. Blue boxes indicate pal II, ssPurines, and deletion/substitutions in pal II. Data are an average of at least three independent experiments. **(B)** Predicted structure of the wild type and mutant SL4 domain in the SHAPE-constrained RNA model by RNAstructure **(C)** The RNAstructure predicted site of interaction (point of dimerization) between two wild type gRNAs. The central 6 nt of pal II are shown in blue.

**Figure 7:**
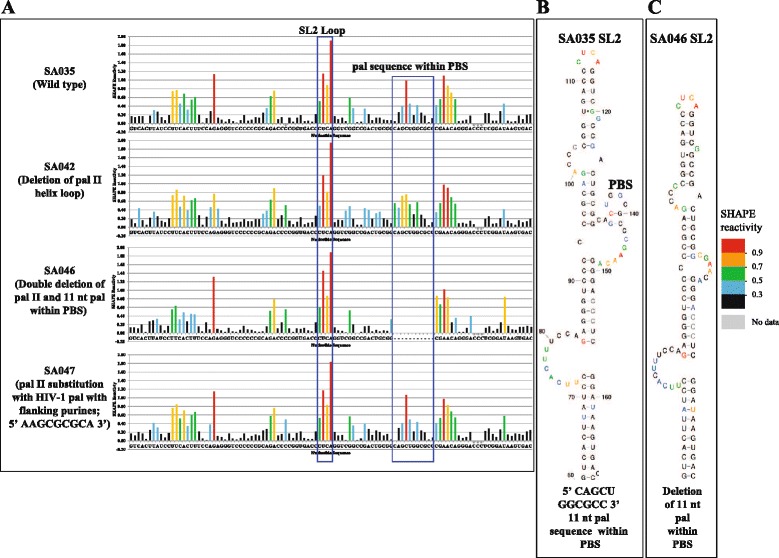
**SHAPE and structural analyses of the SL2 domain of the pal II and PBS pal mutants. (A)** The SHAPE reactivity of the wild type and mutant RNAs are presented as color-coded histograms. The blue boxes highlight the SL2 loop sequences and the 11 nt PBS pal. **(B)** The SHAPE-validated wild type SA035 SL2 domain structure and the nucleotides are color-coded according to SHAPE reactivity. **(C)** The mutant SA046 SL2 structural motif is shown after being constrained by SHAPE data.

In order to test whether the dimerization, packaging and propagation data could be explained by the effects of mutations in pal II and PBS pal on MMTV RNA secondary structure, we performed SHAPE analyses on these mutants in dimer buffer, as described. Under this condition, the wild type RNA (SA035) was largely dimeric when electrophoresis was conducted in TBM buffer, as can be observed in Figures 2, 3, and 5, thus allowing easy interpretation of results. Figure 6 focuses on the SL4 region (~ nt 250-303, encompassing pal II), while Figure 7 on the PBS region, respectively. Overall, no major structural changes could be observed for any of the mutants compared to the wild type. Specifically, in the wild type RNA (SA035) and in the substitution mutant with the HIV-1 pal containing flanking purines (SA047), the two pals displayed limited or no SHAPE reactivity (Figure 6A), consistent with their expected role in RNA dimerization (Figure 5B and Figure 5C). Accordingly, pal II substitution mutants with decreased RNA dimerization displayed high reactivity of at least one nucleotide in the mutated region (SA041, SA044, SA045, Figure 6A). The ssPurines sequence was highly reactive in all RNAs (Figure 6A). Several mutants (SA041, SA044, and SA047) preserved the bifurcated stem loop structure of SL4 (Figure 6B). Surprisingly, SA045 containing a substitution of pal II with non-palindromic sequence adopted a totally different structure in which the mutated sequence was mostly base-paired (Figure 6B). Misfolding of this RNA likely explains why the *trans*-complementary mutants SA044 and SA045 were unable to form heterodimers (Figure 5B and Figure 5C). In the two pal II deletion mutants (SA042 and SA046), SL4 adopted a single hairpin conformation in which most of the nucleotides that formed the bifurcating loop in the wild type structure were base-paired (Figure 6B) explaining the reduction in dimerization observed (Figure 3B and Figure 3C).

Next we analysed the structure of the SL2 domain, which contains the PBS pal, in the wild type and mutant RNAs. Although the PBS is predicted to be unpaired in monomeric RNA, the overlapping PBS pals were weakly reactive (Figure 7A), especially the second pal (5’ GGCGCC 3’), consistent with their potential role in RNA dimerization by forming loose dimers. The reactivity of this region was not significantly affected by deletion or substitutions in pal II (Figure 7A, mutants SA042 and SA047), suggesting the second pal within the PBS could potentially play a role in RNA dimerization (Figure 3B, 3C and Figure 5B, 5C).

### Structural prediction of mutant dimers supports dimerization data

**Figure 8:**
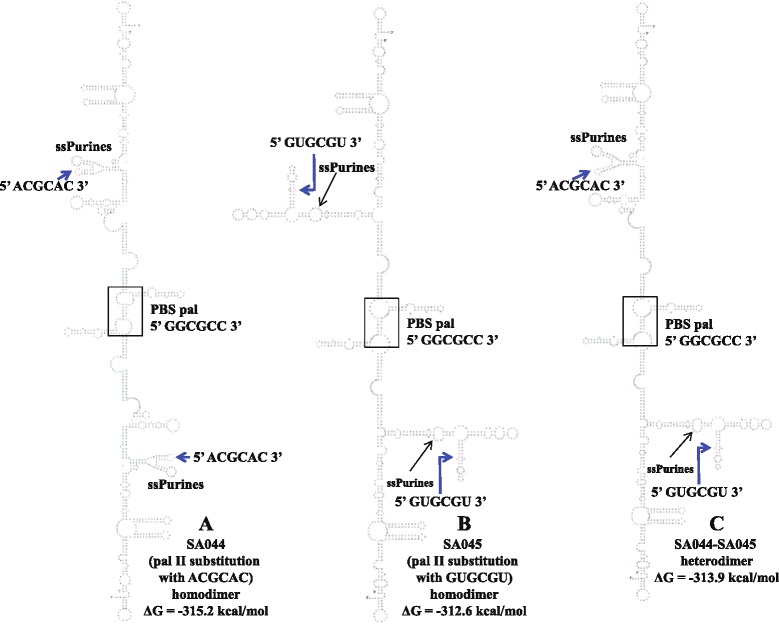
**Structural analysis of pal II** ***trans*** **complementary-mutants.** Predicted homodimer **(A-B)** and heterodimer **(C)** structures of MMTV pal II *trans-*complementary mutants (SA044 and SA045). The dimers were predicted using RNAstructure and the contact point of the sequences (point of dimerization) between two RNAs leading to dimerization are boxed.

To establish a structural basis of the *in vitro* dimerization results, we predicted minimal free energy models of the wild type and mutant MMTV RNA dimers using RNAstructure. This software predicted that wild type RNA dimerizes *via* the pal II sequence (Figure 4A), while deletion of pal II (SA042) induces dimerization *via* the PBS pal (Figure 4B), in agreement with our RNA dimerization and SHAPE data (Figure 3, Figure 5, Figure 6, and Figure 7). The predicted structure of SA051 (deletion of 11 nt pal sequence within PBS), revealed that the native structure (like wild type homodimer) was restored except for the loss of the PBS loop (Figure 4C) which could in part explain its ability to dimerize though much less efficiently when compared to the wild type (Figure 3B and Figure 3C). The mutant with simultaneous deletions of pal II and the PBS pal (SA046) was predicted to dimerize *via* pal III (Figure 4D), but according to our experimental data, this mutant RNA dimerized weakly (Figure 3B and Figure 3C). Taken together, this data suggests that such intermolecular predicted interaction utilizing pal III is unlikely to occur *in vivo. Trans* complementary substitution mutants with non-palindromic sequences in place of pal II (SA044 and SA045) were predicted to homodimerize *via* the PBS pal (Figure 8A and Figure 8B). Surprisingly and in good agreement with our experimental data (Figure 5), these two mutant RNAs were predicted to form heterodimers *via* the PBS pals rather than *via* their *trans*-complementary mutated sequences (Figure 8C).

### Additional mutational analysis of pal II, PBS pal, and pal III confirms the primary role of pal II in MMTV gRNA dimerization

**Figure 9:**
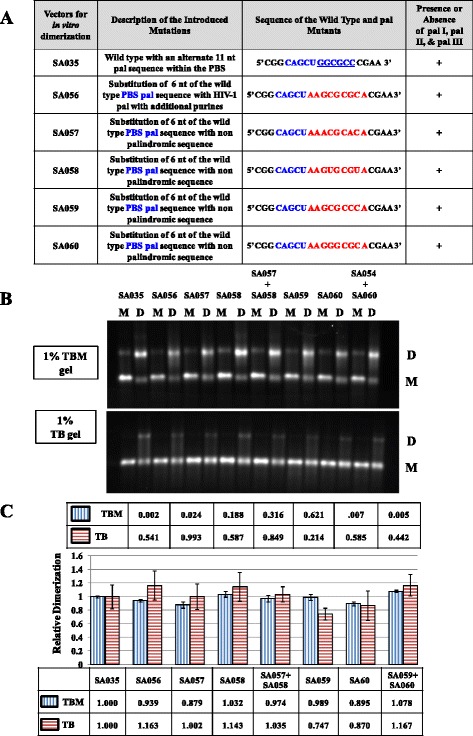
**Further mutational analysis of PBS pal towards MMTV gRNA dimerization. (A)** Description of the PBS pal mutants. **(B)** Typical RNA dimerization in TBM and TB gels of wild type and mutant MMTV RNAs. M: monomer lane or monomer conformer; D: dimer lane or dimer conformer. **(C)** Quantification of the relative RNA dimerization levels (see table below the histogram). Table above the histogram represent the *P* values of the respective mutants in TBM and TB gels. The histograms represent data from at least three independent experiments (± SD).

Results presented in Figure 3 suggested that in addition to pal II, the PBS pal may also play a role in gRNA dimerization since its deletion in SA051 affected gRNA dimerization by ~60%. Structure-prediction analysis further revealed that in the absence of pal II, the point of contact between the two RNAs involved a stretch of palindromic nucleotides (5’ GGCGCC 3’) within the PBS pal. To analyze the role of these 6 nucleotides in PBS pal in RNA dimerization, several substitution mutations were introduced in SA035, the wild type vector (Figure 9A). SA056 replaced the 6 nucleotides of PBS pal with the HIV-1 pal and its flanking purines, while mutants SA057-SA060 replaced the 6 nts PBS pal with non-palindromic *trans* complementary sequences containing additional flanking purines similar to the ones incorporated in SA047 (Figure 5A). Test of these mutants in the *in vitro* dimerization assay revealed that none of the mutations affected dimerization significantly (Figure 9B and Figure 9C). The *trans* complementary mutants SA057- SA060 abrogated the palindromic nature of PBS pal; however, they did not significantly affect dimerization when incubated individually (Figure 9B and Figure 9C). When RNAs from *trans* complementary mutant pairs SA057 + SA058 and SA059 + SA060 were incubated together to initiate the intermolecular interactions owing to the *trans* complementary nature of the substituted sequences, these also showed dimerization to the wild type levels (Figure 9B and Figure 9C). Taken together, these results suggest that PBS pal is unlikely to be the primary point of contact between the two gRNAs to augment efficient MMTV dimerization. They also suggest that the effect of the PBS pal deletion on RNA dimerization (mutant SA051; Figure 3B, Figure 3C,) is indirect. Thus, in agreement with earlier results, these observations argue that pal II acts as the major point of contact, facilitating MMTV gRNA dimerization.

**Figure 10:**
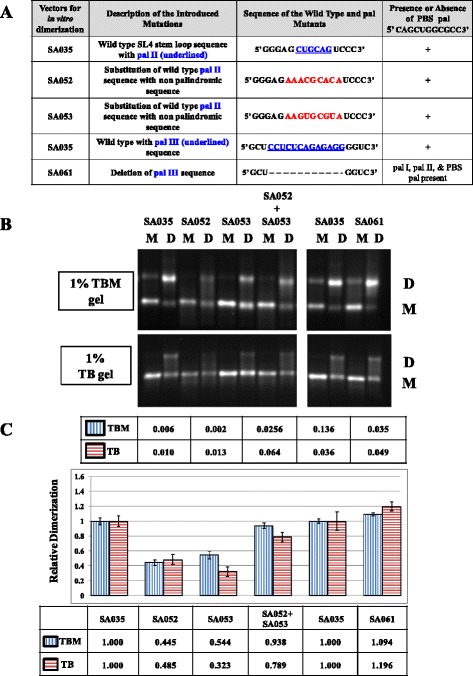
**Further mutational analysis of pal II and pal III towards MMTV gRNA dimerization. (A)** Description of the pal II and pal III mutants. **(B)** Typical RNA dimerization in TBM and TB gels of wild type and mutant MMTV RNAs. M: monomer lane or monomer conformer; D: dimer lane or dimer conformer. **(C)** Quantification of the relative RNA dimerization levels (see table below the histogram). Table above the histogram represent the *P* values of the respective mutants in TBM and TB gels. The histograms represent data from at least three independent experiments (± SD).

Consistent with these results, substitution of pal II with HIV-1 pal containing additional flanking purines, SA047, restored dimerization to the wild type levels (Figure 5C). Furthermore, substitution of pal II with *trans* complementary mutations significantly reduced RNA dimerization when incubated individually (see SA044 + SA045 in Figure 5C), further confirming the role of pal II in RNA dimerization. However, contrary to our expectations, when RNAs from these two *trans* complementary mutants were incubated together, they failed to restore dimerization (Figure 5C). This could in part be explained by the lack of purine residues that have been shown to be crucial for the stability of the RNA dimer [26]. Therefore, we introduced two other substitution mutations containing the exact sequences as in the pal II *trans* complementary mutants, SA044 (5’ ACGCAC 3’) and SA045 (5’GUGCGU 3’) containing flanking purines (highlighted in bold letters) not present in SA044 or SA045, resulting in SA052 (5’ **AA**ACGCAC**A** 3’) and SA053 (5’**AA**GUGCGU**A** 3’) (Figure 10A). As expected, *in vitro* dimerization of these mutant RNAs was greatly affected when they were incubated individually (*P* = 0.006 and 0.002 for SA052 and SA053, respectively (Figure 10B and Figure 10C). On the other hand, when these *trans* complementary mutant RNAs were incubated together, the RNA dimerization was restored to the wild type levels (SA052 + SA053 in Figure 10B and Figure 10C). These results not only confirm that pal II plays a major role in initiating intermolecular interactions between the two RNAs, but also validate the importance of flanking purines in stabilizing RNA dimers mediated by the HIV-1 pal [26].

Finally, structural analysis of the double deletion mutant (SA046; pal II and PBS pal) predicted that this mutant RNA could homodimerize via pal III (Figure 4D). Since dimerization was found to be abrogated for this mutant (Figure 3C), it is unlikely that these dimers are being formed during MMTV replication. To test this experimentally, we created a mutant in which pal III was deleted (SA061; Figure 10A). Test of this mutant revealed that deletion of pal III did not have any effect on dimerization (Figure 10B and Figure 10C), suggesting pal III has no role in MMTV RNA dimerization. Overall, these results suggest that pal II is the primary RNA dimerization determinant in MMTV.

## Discussion

In an attempt to enhance our understanding of the gRNA dimerization and packaging processes during MMTV life cycle, we first validated the predicted structure of MMTV packaging signal RNA employing SHAPE. The SHAPE-validated structure was similar, but not identical to the Mfold-predicted structure. An important feature revealed by SHAPE was a U5/Gag LRI, which was also supported by phylogeny and *in silico* RNA structure predictions (Figure 1, Additional files 1 and 2). The U5/Gag LRI anchors the overall structure of MMTV packaging signal RNA and is consistent with an earlier report showing that the MMTV packaging sequence extends into *gag* [10]. Similar LRIs exist in the packaging signal RNAs of a number of retroviruses [21,23,56-59] and in several cases, disruption of LRIs have been shown to affect RNA dimerization and packaging [33,57,58]. In addition to LRI, SHAPE validated the existence of the SL2 and SL4 domains. The SL2 domain contains the PBS and the pal I sequence, and a similar large SL2 domain is found in a number of other retroviral packaging signal RNAs [8,21,23,56,60]. The bifurcated SL4 hairpin contains a pal helix loop (pal II) and an ssPurines loop (Figure 1). Both SL2 and SL4 structures are conserved in all the 8 MMTV strains studied (Additional files 1 and 2).

The presence of a stretch of purines in the packaging sequences on retroviral gRNA has been proposed to facilitate RNA packaging by functioning as a potential NC binding site [11,26,61,62]. Thus, it is interesting to note that the bifurcated SL4 domain contains a purine-rich loop (5’ GGAGAAGAG 3’) adjacent to the pal II helix loop (Figure 1), reminiscent to the situation found in MPMV [8,21]. In the case of MPMV, genetic and structure-prediction analyses have suggested that either ssPurines or its partial repeat base-paired sequence in an adjacent region plays a crucial role in gRNA packaging, possibly by functioning as a NC binding site [8]. Therefore, it will be interesting to investigate the role of ssPurines in MMTV gRNA packaging.

Retroviral gRNA dimerization has been suggested as a prerequisite for packaging into the budding virions (reviewed in [1-6,63]). This process is usually initiated by conserved palindromic sequences that initiate kissing loop interactions [3,6,21,23,25,29-33,55-57,64,65]. Nucleotide sequence and structural analyses of MMTV packaging signal RNA initially revealed four pal sequences (pal I, pal II, PBS pal, and pal III, Figure 1). Our mutational analysis clearly indicates that neither pal I (Figure 2) nor pal III (Figure 10) is involved in RNA dimerization and packaging. Nevertheless, pal I mutants had significant propagation defects (Figure 2), suggesting that mutations close to the PBS might affect annealing of the primer tRNA or initiation of reverse transcription itself. On the other hand, initial mutational analysis suggested that both pal II and PBS pal play a role in MMTV RNA dimerization (Figure 3 and Figure 5). Indeed, mutations in pal II (SA042) as well as PBS pal (SA051) reduced RNA dimerization by 2-3-folds, while deleting both pal II and PBS pal (SA046) resulted in nearly a complete abrogation of dimerization (Figure 3). However, additional PBS pal mutants (Figure 9) showed that unlike PBS pal deletion (mutant SA046; Figure 3B, 3C), substitutions in PBS pal had no effect on RNA dimerization, even when the wild type sequence was replaced by a non-palindromic sequence (mutants SA057, SA058, SA059 and SA060; Figure 9). These results indicate that the PBS pal does not mediate intermolecular interactions by direct base pairing and strongly suggest that the effect of the PBS pal deletion on RNA dimerization is indirect. Interestingly, deletion of either pal II or the PBS pal had a pronounced effect on RNA packaging (even though the effect of pal II deletion was more dramatic on packaging; Figure 3), suggesting that the MMTV Gag precursor initially recognizes an RNA dimer with two points of contact.

A classical way of demonstrating the existence of any intermolecular interaction between viral RNAs is by using *trans*-complementary mutants [21,53-55,66]. Our initial attempts to restore RNA dimerization using *trans*-complementary pal II mutants were not successful (Figure 5). This unexpected result could however be explained by misfolding of one of the mutants (SA045; Figure 6B and Figure 8B). These two mutants were predicted to form heterodimer *via* the PBS pal rather than *via* their *trans* complementary substituted sequences (Figure 8C). Previous work with HIV-1 that showed that purines flanking the palindromic sequence can enhance the stability of the RNA kissing-loop dimer [26] led us to design additional *trans*-complementary mutants with such purines (Figure 10, mutants SA052 and SA053). While dimerization of these RNA mutants was severely affected when they were incubated alone, they dimerized in *trans* to wild type levels, demonstrating that pal II mediates MMTV RNA dimerization by direct base pairing between two genomic RNA molecules. Along the same lines, results obtained with mutant SA047 (in which pal II was replaced by the HIV-1 pal with the flanking purines) displayed wild type RNA dimerization levels; however, its RNA packaging and propagation were abolished (Figure 5), suggesting that the pal II hairpin could also be involved in RNA-protein interactions during genome encapsidation, perhaps at the primary sequence level. The overall 3D structure of the packaging domain would thus probably be important for Gag binding. This could explain why deletion of 5’ UTR sequences forming the basal part in the SHAPE-validated structure of SL2 abrogated MMTV gRNA packaging and propagation [10].

## Conclusions

Results presented in this study validate the higher order structural features of the MMTV packaging signal RNA using the SHAPE methodology, which includes LRI involving complementary sequences from U5 and the *gag* (U5/Gag LRI), ssPurines, and four palindromic sequences (Figure 1). Employing a combination of genetic, biochemical, and structural prediction approaches, we further show that the MMTV genome uses pal II as the primary point of contact, by likely functioning as the DIS, to initiate intermolecular interactions augmenting efficient gRNA dimerization, and leading to its encapsidation in the assembling virus particles (Figure 3 and Figure 4, and Figure 10). SHAPE analyses of pal II mutants further support its role in MMTV gRNA dimerization (Figure 6). These results are in agreement with earlier studies on determinants of MMTV gRNA packaging, and further reveal important functional relationships between gRNA dimerization and packaging processes, suggesting that MMTV prefers a dimeric genome as a packaging substrate.

## Methods

### Structural analyses

The secondary structure of the 5’ region of the MMTV gRNA containing either 120 nt of *gag* (432 nt of the 5’ end from R) or 400 nt of *gag* (containing 712 nt of the 5’ end from R) were predicted using the Mfold server [67,68]. The SHAPE reactivity data of the first 432 nt region was applied in RNAstructure software (version 5.3) [69] as constraints to develop the final structural model. The Mfold-predicted and SHAPE-validated structures were redrawn with the XRNA software (http://rna.ucsc.edu/rnacenter/xrna/xrna.html). MMTV nucleotide positions refer to HYBMTV a molecular clone created by Shackleford and Varmus [70].

### Phylogenetic analyses

Nucleotides 1-432 of the gRNA of different strains of MMTV were aligned using Clustal Omega [71,72] and a consensus or “conservation annotation” structure for these strains was predicted using RNAalifold server [73,74]. The accession numbers for different MMTV strains used for sequence alignment are AF228550.1 (*mtv-*1) [75], L37517.1 (*mtv*-6) [76], AF033807.1 [77], M15122.1 (BR6) [78], D16249.1 (JYG) [78], AF228551.1 (C3H/HeJ) [75], X00018.1 (GR) [79] and AF228552.1 (C3H) [75].

### Construction of plasmids

The 5’ end of MMTV gRNA corresponding to nt 1-712 was amplified by polymerase chain reaction (PCR) using the MMTV sub-genomic transfer vector DA024 [45] as the template and the sense (S) OTR 984 (5’ CCC*AAGCT****T*AATACGACTCACTATAGGG**CAACAGTCCTAATATTCACG 3’) and the antisense (AS) OTR 985 (5’ AAA*CCCGGG*TTCCCCTGGTCCCATAAG 3’) primers. These primers include the T7 promoter sequence (in bold) at the 5’ end along with flanking restriction sites (*HindIII* and *XmaI/SmaI*, shown in italics). The *HindIII* and *XmaI* sites were utilized to introduce the MMTV sequence into a pUC-based cloning vector (pIC19R) [80]. Introduction of these sequences (from OTRs 984 and 985) resulted in the addition of GGG and CCC complementary sequences at the ends of the RNA which created base pairing potential; however, folding of the 5’ end of the MMTV genome containing these artificially-introduced complementary sequences in Mfold did not predict base pairing among these sequences nor alter the overall RNA secondary structure of this region (data not shown). The sequence of the resulting clone, SA035, was confirmed by sequencing. SA035 (containing 400 nt of *gag*) was also used as the wild type clone for *in vitro* gRNA dimerization assays.

A number of mutations, including deletions, substitutions and substitution containing heterologous *trans*-complementary sequences, were introduced in pal I (5’ GUCGGCCGAC 3’), pal II (5’ CUGCAG 3’), PBS pal (5’ CAGCUGGCGCC 3’), and pal III (5’ CCUCUCAGAGAGG 3’) through splice overlap extension (SOE) PCR as described previously [8,21,33,81]. Briefly, in round one, the MMTV sub-genomic vector DA024 was amplified in two separate PCRs using S and AS primers generating products having overlapping complementary sequences. In round two, the products from round one PCRs, which anneal *via* their overlapping complementary sequences, were amplified using outer primers OTR 984 (S) and OTR 985 (AS), generating a final product containing the desired mutation(s). The sequences of S and AS primers used to introduce mutations are provided in Additional file 4, along with the clone names and the necessary description. Some mutations were also cloned in the MMTV subgenomic transfer vector, DA024, to study the effect of these mutations on RNA packaging and propagation. All clones were confirmed by sequencing. Further details of cloning can be obtained from the authors upon request.

### *In vitro* transcription assay

After linearization with the *SmaI* restriction enzyme, the wild type (SA035) or mutant (SA031-SA033, SA041-SA045, SA046, SA047 and SA051) clones were transcribed *in vitro* to generate the RNAs to be used in dimerization assays, as previously described [82]. Following DNase treatment, RNA extraction and purification, the RNAs were concentrated using Amicon Ultra-4 10 K devices (Millipore) and their concentration was determined using nanodrop (ThermoScientific), as described previously [21]. Finally, the quality of the purified RNAs was checked by denaturing polyacrylamide gel electrophoresis.

### SHAPE methodology

SHAPE [46-48] was performed on *in vitro* transcribed RNAs using benzoyl cyanide (BzCN) following the protocol described recently [21]. Briefly, RNAs were modified in the dimer buffer (final concentrations: 50 mM sodium cacodylate, (pH 7.5), 300 mM KCl and 5 mM MgCl_2_) in the presence of 2 μg total yeast tRNA (Sigma Aldrich). The dimer buffer was used for the SHAPE analysis to help identify points of contact involved in the dimerization process. The modified RNAs were reverse transcribed using two sets of AS primers: OTRs 9, 10, 11 and 12 (5’ AACAGATTTGGCTTCTGCGG 3’; MMTV nt 614-633) and OTRs 13, 14, 15 and 16 (5’ AGTTTCTGCCCTTTTGAGCC 3’; MMTV nt 325-344), labelled with VIC, FAM, NED, or PET, respectively. These two sets of primers enabled the revere transcription of the entire RNA sequence except for a few nucleotides at the 3’ end due to primer binding. The primer extension products were loaded onto an Applied Biosystems 3130xl genetic analyzer and the electropherograms were analysed with the SHAPEfinder [49,83]. The SHAPE reactivities were determined and normalized as described earlier [21,49]. The normalized SHAPE reactivity data from at least 3-6 independent experiments (submitted as Additional file 5) were used as constraints to validate the RNA secondary structure of the MMTV packaging signal with RNAstructure (version 5.3) [69] and also to determine the effects of pal mutations on the overall higher order structure.

### *In vitro* dimerization assay

*In vitro* dimerization assays of wild type and mutant MMTV RNAs were performed as described previously [21,82]. Briefly, 300 nM RNAs were heated at 90°C and incubated in ice for 2 min followed by a 37°C incubation for 30 min in either the dimer (final concentrations: 50 mM sodium cacodylate, pH 7.5; 300 mM KCl; 5 mM MgCl_2_) or monomer (final concentrations: 50 mM sodium cacodylate, pH 7.5; 40 mM KCl; 0.1 mM MgCl_2_) buffer. The samples were divided equally and analysed by electrophoresis in TBM (50 mM Tris base, 45 mM boric acid, 0.1 mM MgCl_2_) and TB (50 mM Tris base, 45 mM boric acid) ethidium bromide prestained agarose gels at 4°C or 20°C, respectively. Density of the dimeric and monomeric species was quantitated using Quantity One software, and percentage of dimerization was calculated and expressed as dimerization relative to the wild type for each mutant, as described previously [21]. For the *in vitro* dimerization assay of *trans* complementary mutants, equal amounts (150 nM) of *in vitro* transcribed RNAs from both the mutants (for a total of 300 nM) were incubated together before electrophoresis, as described above.

### *In vivo* genetic complementation assay

The *in vivo* genetic *trans*-complementation assay used two expression plasmids, JA10 and MD.G, to provide the structural and enzymatic genes from MMTV Gag/Pol and vesicular stomatitis virus (VSV-G) envelope regions, respectively [45,84]. A third plasmid (MMTV sub-genomic transfer vector-DA024) containing minimum *cis*-acting sequences required for RNA packaging, reverse transcription, and integration, served as a source of the packageable vector RNA [45]. Design and rationale of the MMTV 3-plasmid genetic complementation assay developed earlier by our group is schematically shown in Additional file 3 [45]. Briefly, the particles produced by JA10 and MD.G allow packaging of the transfer vector RNA by virtue of the presence of the conventional packaging signal (ψ) on the MMTV transfer vector (DA024). The pal I, pal II, and PBS pal mutations introduced into the T7 promoter-based vector were further cloned into the MMTV sub-genomic transfer vector, DA024, and tested in this assay to monitor their effects on gRNA packaging and propagation.

Human embryonic kidney (HEK) 293T producer cells were transfected using the calcium phosphate transfection protocol to produce virus particles containing the wild type or mutated RNAs, as described previously [10,45]. The luciferase expression vector, pGL3 (Promega), was used as an internal control to measure transfection efficiency, as described earlier [8-10,33]. Supernatants containing viral particles were collected and spun down at 4000 rpm on a table top centrifuge for 10 min. A portion of the supernatant was used to infect the human cervical cancer cell line (HeLaT4) to determine the transduction efficiency by selection with medium containing 200 μg/ml of hygromycin B (Hyclone). Cells transduced with virus particles containing the packageable transfer vector RNA express the marker *Hyg*^*r*^ gene, resulting in colonies which are measured as colony forming units (CFU/ml). The CFU/ml values were then normalized to the transfection efficiency. The normalized propagation efficiencies were then divided by the wild type values to represent the propagation of the mutants relative to the wild type (Relative CFU/ml).

### Nucleocytoplasmic fractionation, isolation of RNA, and cDNA preparation

Cells from the transfected cultures were fractionated into nuclear and cytoplasmic fractions as described previously [9,85]. Cellular RNAs from the cytoplasmic fractions and packaged viral RNA were isolated from the pelleted viral particles using Trizol and Trizol LS reagents, respectively (Invitrogen), as described earlier [9,85]. The extracted RNAs were amplified for 30 cycles by conventional PCR using vector-specific primers OTR 671(S) or and OTR 672 (AS) (Additional file 4) after DNase (Promega) treatment to ensure that there was no contaminating DNA present in the RNA samples. The PCR conditions were as follows: denaturation for 2 min at 94°C, followed by 30 cycles of 45 sec at 90°C, annealing for 45 sec at 60°C and extension for 45 sec at 72°C followed by incubation at 4°C. The DNase-treated cytoplasmic and viral RNA samples were reverse transcribed into cDNA and PCR amplified to determine the quality of the cDNA samples. To determine the integrity of the nuclear membrane during cytoplasmic RNA extraction, a multiplex PCR was performed on the cDNAs prepared from the cytoplasmic RNA fractions using OTR 582 (S) and OTR 581 (AS) (Additional file 4) designed to amplify unspliced β-actin mRNA [9,85]. Amplifications of the cDNAs using primers/competimer for 18S ribosomal RNA (18S Quantum competimer control, Ambion) was also performed as an ancillary control for the presence of amplifiable cDNA in the multiplex PCRs [9,85].

### Real time quantitative PCR (qPCR) for transfer vector RNA packaging efficiency

To determine the packaging efficiency of the transfer vector RNA, quantitative qPCR was performed on the cDNA prepared from the cytoplasmic and viral RNAs as described earlier [10]. The endogenous control for qPCR was β-actin (Human β-actin Endogenous Control assay, VIC⁄MGB probe, Applied Biosystems). The primers and probes for MMTV transfer vector RNAs in the FAM-based Applied Biosystems custom expression assay anneal at a 68 nt region in the MMTV U5 (nt 1192-1259) common to wild type and mutant RNAs. The relative amplification efficiencies of the MMTV and β-actin expression assays were determined to be 0.0126, thus, validating the assays for quantitative PCR analysis using the ΔΔCt method [10]. Equal amounts of viral and cytoplasmic cDNA were tested in triplicates. The amplification conditions used were as follows: Uracil-N-glycosylase incubation 2 min at 50°C, denaturation for 10 min at 95°C, followed by 40 cycles of denaturation and annealing/extension steps at 95°C for 15 sec and 60°C for 1 min. The relative packaging efficiencies were derived following a modification of our earlier described method [10]. Briefly, the raw Ct (cycle threshold) values for the MMTV transfer vector were normalized by the Ct values of the β-actin endogenous control. The normalized values were then calculated relative to the mock sample (relative quantification, RQ) that was transfected with all the plasmids except the transfer vector plasmid forming the empty virus particles without any packaged RNA. For final relative packaging efficiency calculations, the relative viral RQ values were divided by the relative cytoplasmic RQ values, and the resulting values were normalized to the transfection efficiencies. The final values are presented relative to the wild type.

### Statistical analysis

The standard paired, two-tailed Student’s *t-*test was performed between the wild type and mutant clones to determine statistically significant differences. For *in vitro* dimerization assays, and *in vivo* packaging and propagation studies, a *p* value of ≤0.05 was considered significant.

## Additional files

Additional file 1
**Phylogenetic conservation of the sequences of major structural motifs of MMTV packaging signal RNA in different strains.** (A) Clustal Omega sequence alignment of eight different strains of MMTV packaging signal RNA. Sequences of the major structural motifs are highlighted in different colors, whereas the 11 nt pal sequence within PBS is boxed. The accession numbers for the MMTV strains are provided in the Materials and Methods. (B) Sequence conservation superimposed on the SHAPE validated structure. The nucleotides are color-coded based on their conservation level, ranging from <75 to 100%. Additional file 2
**Consensus RNA secondary structure prediction of MMTV packaging signal RNA using sequences from eight different strains.** Accession numbers of MMTV strains are the same as mentioned in materials and methods section. The sequences of the 8 MMTV strains were aligned using Clustal Omega and the aligned sequences were used as input in the RNAalifold server [73,74] to predict the consensus or “conservation annotation” structure. The number/color scale on the x-axis indicates base pairing conservation with 1 (red color) representing completely conserved base pairing to 6 (violet color) representing 6 different types of base pairings at that position among the various strains (see Methods for details). The y-axis represents the incompatibility score based on non-Watson base pairing within each strain. The number (0-2) or color gradation (dark to very pale) represents increasing number of strains showing incompatible (non-Watson) base pairing within the structure of the various strains. Thus, a dark red color denotes “highest” conservation of base pairings, while a light violet color represents “least” conservation of base pairing among different strains of MMTVs. Additional file 3
**Schematic representation of the design and rationale of three plasmid trans complementation assay for studying MMTV gRNA packaging and propagation developed earlier [** 45 **].** The pseudotyped particles produced following transfection of human embryonic kidney (HEK) 293T cells with MMTV Gag/Pol expression plasmid (JA10) and VSV-G envelope expression plasmid (MD.G) allows packaging of the transfer vector RNA by virtue of the presence of the packaging signal (ψ) on the MMTV transfer vector (DA024). Virus particles produced following transfection contain the packaged transfer vector RNA (DA024) and are used to quantitate RNA packaging by real time PCR or to infect target cells to monitor RNA propagation. Additional file 4
**Table describing primers used for cloning, sequencing, and conventional and real time PCR.**
Additional file 5
**Normalized SHAPE reactivity data from at least three to six independent experiments.**

## References

[1] D’Souza V, Summers MF (2005). How retroviruses select their genomes. Nat Rev Microbiol.

[2] Johnson SF, Telesnitsky A: **Retroviral RNA dimerization and packaging: the what, how, when, where, and why.** *PLoS Pathog* 2010, **6:**e1001007. 10.1371/journal.ppat.1001007 PMC2951377 20949075

[3] Lever AML (2007). HIV RNA packaging. Adv Pharmacol.

[4] Lu K, Heng X, Garyu L, Monti S, Garcia EL, Kharytonchyk S, Dorjsuren B, Kulandaivel G, Jones S, Hiremath A, Divakaruni SS, LaCotti C, Barton S, Tummillo D, Hosic A, Edme K, Albrecht S, Telesnitsky A, Summers MF (2011). NMR detection of structures in the HIV-1 5’-leader RNA that regulate genome packaging. Science.

[5] Paillart JC, Shehu-Xhilaga M, Marquet R, Mak J (2004). Dimerization of retroviral RNA genomes: An inseparable pair. Nat Rev Microbiol.

[6] Russell RS, Liang C, Wainberg MA: **Is HIV-1 RNA dimerization a prerequisite for packaging? Yes, no, probably?** *Retrovirology* 2004, **1:**23. 10.1186/1742-4690-1-23 PMC516451 15345057

[7] Abd El-Wahab EW, Smyth RP, Mailler E, Bernacchi S, Vivet-Boudou V, Hijnen M, Jossinet F, Mak J, Paillart JC, Marquet R: **Specific recognition of the HIV-1 genomic RNA by the Gag precursor.** *Nat Commun* 2014, **5:**4304. doi:10.1038/ncomms5304. 10.1038/ncomms5304 24986025

[8] Jaballah SA, Aktar SJ, Ali J, Phillip PS, Al Dhaheri NS, Jabeen A, Rizvi TA (2010). A G-C rich palindromic structural motif and a stretch of single stranded purines are required for optimal packaging of Mason-Pfizer monkey virus (MPMV) genomic RNA. J Mol Biol.

[9] Mustafa F, Ghazawi A, Jayanth P, Phillip PS, Ali J, Rizvi TA (2005). Sequences intervening between the core packaging determinants are dispensable for maintaining the packaging potential and propagation of feline immunodeficiency virus transfer vector RNAs. J Virol.

[10] Mustafa F, Al Amri D, Al Ali F, Al Sari N, Al Suwaidi S, Jayanth P, Philips PS, Rizvi TA: **Sequences within both the 5’ UTR and Gag are required for optimal in vivo packaging and propagation of mouse mammary tumor virus (MMTV) genomic RNA.** *PLoS One* 2012, **7:**e47088. 10.1371/journal.pone.0047088 PMC3473059 23077548

[11] Moore MD, Hu WS (2009). HIV-1 RNA dimerization: It takes two to tango. AIDS Rev.

[12] Al Dhaheri NS, Phillip PS, Ghazawi A, Ali J, Beebi E, Jaballah SA, Rizvi TA: **Cross-packaging of genetically distinct mouse and primate retroviral RNAs.** *Retrovirology* 2009, **6:**66. 10.1186/1742-4690-6-66 PMC2723071 19602292

[13] Al Shamsi IR, Al Dhaheri NS, Phillip PS, Mustafa F, Rizvi TA (2011). Reciprocal cross-packaging of primate lentiviral (HIV-1 and SIV) RNAs by heterologous non-lentiviral MPMV proteins. Virus Res.

[14] Motomura K, Chen J, Hu WS (2008). Genetic recombination between human immunodeficiency virus type 1 (HIV-1) and HIV-2, two distinct human lentiviruses. J Virol.

[15] Moore MD, Fu W, Nikolaitchik O, Chen J, Ptak RG, Hu WS (2007). Dimer initiation signal of human immunodeficiency virus type 1: its role in partner selection during RNA copackaging and its effects on recombination. J Virol.

[16] Parveen Z, Mukhtar M, Goodrich A, Acheampong E, Dornburg R, Pomerantz RJ (2004). Cross-packaging of human immunodeficiency virus type 1 vector RNA by spleen necrosis virus proteins: construction of a new generation of spleen necrosis virus-derived retroviral vectors. J Virol.

[17] Rizvi TA, Panganiban AT (1993). Simian immunodeficiency virus RNA is efficiently encapsidated by human immunodeficiency virus type 1 particles. J Virol.

[18] White SM, Renda M, Nam NY, Klimatcheva E, Zhu Y, Fisk J, Halterman M, Rimel BJ, Federoff H, Pandya S, Rosenblatt JD, Planelles V (1999). Lentivirus vectors using human and simian immunodeficiency virus elements. J Virol.

[19] Yin PD, Hu WS (1997). RNAs from genetically distinct retroviruses can copackage and exchange genetic information in vivo. J Virol.

[20] Miyazaki Y, Miyake A, Nomaguchi M, Adachi A (2011). Structural dynamics of retroviral genome and the packaging. Frontiers in Microbiology.

[21] Aktar SJ, Jabeen A, Ali LM, Vivet-Boudou V, Marquet R, Rizvi TA (2013). SHAPE analysis of the 5’ end of the Mason-Pfizer monkey virus (MPMV) genomic RNA reveals structural elements required for genome dimerization. RNA.

[22] Clever JL, Wong ML, Parslow TG (1996). Requirements for kissing-loop-mediated dimerization of human immunodeficiency virus RNA. J Virol.

[23] Kenyon JC, Tanner S, Legiewicz M, Phillip PS, Rizvi TA, LeGrice S, Lever AM (2011). SHAPE analysis of the FIV leader RNA reveals a structural switch potentially controlling viral packaging and genome dimerization. Nucleic Acids Res.

[24] Paillart JC, Marquet R, Skripkin E, Ehresmann B, Ehresmann C (1994). Mutational analysis of the bipartite dimer linkage structure of human immunodeficiency virus type 1 genomic RNA. J Biol Chem.

[25] Paillart JC, Berthoux L, Ottmann M, Darlix JL, Marquet R, Ehresmann B, Ehresmann C (1996). A dual role of the putative RNA dimerization initiation site of human immunodeficiency virus type 1 in genomic RNA packaging and proviral DNA synthesis. J Virol.

[26] Paillart JC, Westhof E, Ehresmann C, Ehresmann B, Marquet R (1997). Non-canonical interactions in a kissing loop complex: the dimerization initiation site of HIV-1 genomic RNA. J Mol Biol.

[27] Hussein ITM, Ni N, Galli A, Chen J, Moore MD, Hu WS (2010). Delineation of the preferences and requirements of the human immunodeficiency virus type 1 dimerization initiation signal by using an in vivo cell-based selection approach. J Virol.

[28] Baig TT, Lanchy JM, Lodmell JS (2007). HIV-2 RNA dimerization is regulated by intramolecular interactions *in vitro*. RNA.

[29] Berkhout B, van Wamel JL (1996). Role of the DIS hairpin in replication of human immunodeficiency virus type 1. J Virol.

[30] Lanchy JM, Ivanovitch JD, Lodmell JS (2003). A structural linkage between the dimerization and encapsidation signals in HIV-2 leader RNA. RNA.

[31] Lanchy JM, Lodmell JS (2007). An extended stem-loop 1 is necessary for human immunodeficiency virus type 2 replication and affects genomic RNA encapsidation. J Virol.

[32] Laughrea M, Jetté L, Mak J, Kleiman L, Liang C, Wainberg MA (1997). Mutations in the kissing-loop hairpin of human immunodeficiency virus type 1 reduce viral infectivity as well as genomic RNA packaging and dimerization. J Virol.

[33] Rizvi TA, Kenyon JC, Ali J, Aktar SJ, Phillip PS, Ghazawi A, Mustafa F, Lever AML (2010). Optimal packaging of FIV genomic RNA depends upon a conserved long-range interaction and a palindromic sequence within gag. J Mol Biol.

[34] Polge E, Darlix JL, Paoletti J, Fossé P (2000). Characterization of loose and tight dimer forms of avian leukosis virus RNA. J Mol Biol.

[35] Houzet L, Paillart JC, Smagulova F, Maurel S, Morichaud Z, Marquet R, Mougel M (2007). HIV controls the selective packaging of genomic, spliced viral and cellular RNAs into virions through different mechanisms. Nucleic Acids Res.

[36] Shen N, Jette L, Liang C, Wainberg MA, Laughrea M (2000). Impact of human immunodeficiency virus type 1 RNA dimerization on viral infectivity and of stem-loop B on RNA dimerization and reverse transcription and dissociation of dimerization from packaging. J Virol.

[37] Andersen ES, Jeeninga RE, Damgaard CK, Berkhout B, Kjems J (2003). Dimerization and template switching in the 5’ untranslated region between various subtypes of human immunodeficiency virus type 1. J Virol.

[38] Balakrishnan M, Fay PJ, Bambara RA (2001). The kissing hairpin sequence promotes recombination within the HIV-I 5’ leader region. J Biol Chem.

[39] Chin MP, Rhodes TD, Chen J, Fu W, Hu WS (2005). Identification of a major restriction in HIV-1 intersubtype recombination. Proc Natl Acad Sci.

[40] Ennifar E, Paillart JC, Marquet R, Ehresmann B, Ehresmann C, Dumas P, Walter P (2003). HIV-1 RNA dimerization initiation site is structurally similar to the ribosomal A site and binds aminoglycoside antibiotics. J Biol Chem.

[41] Ennifar E, Paillart JC, Bodlenner A, Walter P, Weibel JM, Aubertin AM, Pale P, Dumas P, Marquet R (2006). Targeting the dimerization initiation site of HIV-1 RNA with aminoglycosides: from crystal to cell. Nucleic Acids Res.

[42] Cardiff RD, Kenney N (2007). Mouse Mammary Tumor Biology: A Short History. Adv Cancer Res.

[43] Ross SR (2010). Mouse mammary tumor virus molecular biology and oncogenesis. Viruses.

[44] Salmons B, Moritz-Legrand S, Garcha I, Günzburg WH (1989). Construction and characterization of a packaging cell line for MMTV-based conditional retroviral vectors. Biochem Biophys Res Commun.

[45] Rizvi TA, Ali J, Phillip PS, Ghazawi A, Jayanth P, Mustafa F (2009). Role of a heterologous retroviral transport element in the development of genetic complementation assay for mouse mammary tumor virus (MMTV) replication. Virology.

[46] Merino EJ, Wilkinson KA, Coughlan JL, Weeks KM (2005). RNA structure analysis at single nucleotide resolution by selective 2’-hydroxyl acylation and primer extension (SHAPE). J Am Chem Soc.

[47] Mortimer SA, Weeks KM (2007). A fast-acting reagent for accurate analysis of RNA secondary and tertiary structure by SHAPE chemistry. J Am Chem Soc.

[48] Mortimer SA, Weeks KM (2009). Time-resolved RNA SHAPE chemistry: quantitative RNA structure analysis in one-second snapshots and at single-nucleotide resolution. Nat Protoc.

[49] Vasa SM, Guex N, Wilkinson KA, Weeks KM, Giddings MC (2008). ShapeFinder: a software system for high-throughput quantitative analysis of nucleic acid reactivity information resolved by capillary electrophoresis. RNA.

[50] Laughrea M, Jetté L (1996). Kissing-loop model of HIV-1 genome dimerization: HIV-1 RNAs can assume alternative dimeric forms, and all sequences upstream or downstream of hairpin 248-271 are dispensable for dimer formation. Biochemistry.

[51] Mikkelsen JG, Lund AH, Kristensen KD, Duch M, Sørensen MS, Jørgensen P, Pedersen FS (1996). A preferred region for recombinational patch repair in the 5’ untranslated region of primer binding site-impaired murine leukemia virus vectors. J Virol.

[52] Oh J, McWilliams MJ, Julias JG, Stephen Hughes H (2008). Mutations in the U5 region adjacent to the primer binding site affect tRNA cleavage by human immunodeficiency virus type 1 reverse transcriptase in vivo. J Virol.

[53] Chen J, Nikolaitchik O, Singh J, Wright A, Bencsics CE, Coffin JM, Ni N, Lockett S, Pathak VK, Hu WS (2009). High efficiency of HIV-1 genomic RNA packaging and heterozygote formation revealed by single virion analysis. Proc Natl Acad Sci U S A.

[54] Jossinet F, Lodmell JS, Ehresmann C, Ehresmann B, Marquet R (2001). Identification of the in vitro HIV-2/SIV RNA dimerization site reveals striking differences with HIV-1. J Biol Chem.

[55] Paillart JC, Skripkin E, Ehresmann B, Ehresmann C, Marquet R (1996). A loop-loop “kissing” complex is the essential part of the dimer linkage of genomic HIV-1 RNA. Proc Natl Acad Sci U S A.

[56] Kenyon JC, Ghazawi A, Cheung WK, Phillip PS, Rizvi TA, Lever AM: **The secondary structure of the 5’ end of the FIV genome reveals a long-range interaction between R/U5 and gag sequence, and a large, stable stem loop.** *RNA* 2008, **14:**2597. 10.1261/rna.1284908 PMC2590967 18974279

[57] Paillart JC, Skripkin E, Ehresmann B, Ehresmann C, Marquet R (2002). *In vitro* evidence for a long range pseudoknot in the 5’-untranslated and matrix coding regions of HIV-1 genomic RNA. J Biol Chem.

[58] Song R, Kafaie J, Laughrea M (2008). Role of the 5’ TAR stem-loop and the U5-AUG duplex in dimerization of HIV-1 genomic RNA. Biochemistry.

[59] Spriggs S, Garyu L, Connor R, Summers MF (2008). Potential intra- and intermolecular interactions involving the unique 5’ region of the HIV-1 5’ UTR. Biochemistry.

[60] Baudin F, Marquet R, Isel C, Darlix JL, Ehresmann B, Ehresmann C (1993). Functional sites in the 5’ region of human immunodeficiency virus type 1 RNA form defined structural domains. J Mol Biol.

[61] Lever AML: **RNA packaging in lentiviruses.** *Retrovirology* 2009, **6:**113.

[62] Zeffman A, Hassard S, Varani G, Lever A (2000). The major HIV-1 packaging signal is an extended bulged stem loop whose structure is altered on interaction with the Gag polyprotein. J Mol Biol.

[63] Nikolaitchik OA, Dilley KA, Fu W, Gorelick RJ, Tai SH, Soheilian F, Ptak RG, Nagashima K, Pathak VK, Hu WS: **Dimeric RNA recognition regulates HIV-1 genome packaging.** *PLoS Pathog* 2013, **9:**e1003249. 10.1371/journal.ppat.1003249 PMC3605237 23555259

[64] Leitner T, Foley B, Hahn B, Marx P, McCutchan F, Mellors J, Wolinsky S, Korber B (Eds): *HIV Sequence Compendium 2005.* Theoretical Biology and Biophysics Group, Los Alamos National Laboratory, NM, LA-UR 06-0680; 2005.

[65] Skripkin E, Paillart JC, Marquet R, Ehresmann B, Ehresmann C (1994). Identification of the primary site of the human immunodeficiency virus type 1 RNA dimerization in vitro. Proc Natl Acad Sci U S A.

[66] Gavazzi C, Yver M, Isel C, Smyth RP, Rosa-Calatrava M, Lina B, Moulès V, Marquet R (2013). A functional sequence-specific interaction between influenza A virus genomic RNA segments. Proc Natl Acad SciUSA.

[67] Mathews DH, Sabina J, Zuker M, Turner DH (1999). Expanded sequence dependence of thermodynamic parameters improves prediction of RNA secondary structure. J Mol Biol.

[68] Zuker M (2003). Mfold web server for nucleic acid folding and hybridization prediction. Nucleic Acids Res.

[69] Reuter JS, Mathews DH: **RNA structure: software for RNA secondary structure prediction and analysis.** *BMC Bioinformatics* 2010, **11:**129. 10.1186/1471-2105-11-129 PMC2984261 20230624

[70] Shackleford GM, Varmus HE (1988). Construction of a clonable, infectious, and tumorigenic mouse mammary tumor virus provirus and a derivative genetic vector. Proc Natl Acad Sci.

[71] Goujon M, McWilliam H, Li W, Valentin F, Squizzato S, Paern J, Lopez R (2010). A new bioinformatics analysis tools framework at EMBL-EBI. Nucleic Acids Res.

[72] Sievers F, Wilm A, Dineen D, Gibson TJ, Karplus K, Li W, Lopez R, McWilliam H, Remmert M, Söding J, Thompson JD, Higgins DG: **Fast, scalable generation of high-quality protein multiple sequence alignments using Clustal Omega.** *Mol Syst Biol* 2011, **7:**539. doi:10.1038/msb.2011.75. 10.1038/msb.2011.75 PMC3261699 21988835

[73] Hofacker IL, Fekete M, Stadler PF (2002). Secondary Structure Prediction for Aligned RNA Sequences. J Mol Biol.

[74] Bernhart SH, Hofacker IL, Will S, Gruber AR, Stadler PF (2008). RNAalifold: improved consensus structure prediction for RNA alignments. BMC Bioinformatics.

[75] Hook LM, Agafonova Y, Ross SR, Turner SJ, Golovkina TV (2000). Genetics of mouse mammary tumor virus-induced mammary tumors: linkage of tumor induction to the gag gene. J Virol.

[76] Cho K, Ferrick DA, Morris DW (1995). Structure and biological activity of the subgenomic Mtv-6 endogenous provirus. Virology.

[77] Petropoulos CJ, Coffin JM (1997). Retroviral taxonomy, protein structure, sequences, and genetic maps. Retroviruses.

[78] Moore R, Dixon M, Smith R, Peters G, Dickson C (1987). Complete nucleotide sequence of a milk-transmitted mouse mammary tumor virus: two frameshift suppression events are required for translation of gag and pol. J Virol.

[79] Fasel N, Buetti E, Firzlaff J, Pearson K, Diggelmann H (1983). Nucleotide sequence of the 5’ noncoding region and part of the gag gene of mouse mammary tumor virus; identification of the 5’ splicing site for subgenomic mRNAs. Nucleic Acids Res.

[80] Marsh JL, Erfle M, Wykes EJ (1984). The pIC plasmid and phage vectors with versatile cloning sites for recombinant selection by insertional inactivation. Gene.

[81] Gibbs JS, Regier DA, Desrosiers RC (1994). Construction and in vitro properties of SIV mac mutants with deletions in “nonessential” genes. AIDS Res Hum Retrovirus.

[82] Marquet R, Baudin F, Gabus C, Darlix JL, Mougel M, Ehresmann C, Ehresmann B (1991). Dimerization of human immunodeficiency virus (type 1) RNA: stimulation by cations and possible mechanism. Nucleic Acids Res.

[83] Wilkinson KA, Gorelick RJ, Vasa SM, Guex N, Rein A, Mathews DH, Giddings MC, Weeks KM: **High-throughput SHAPE analysis reveals structures in HIV-1 genomic RNA strongly conserved across distinct biological states.** *PLoS Biol* 2008, **6:**e96. doi:10.1371/journal.pbio.0060096. 10.1371/journal.pbio.0060096 PMC2689691 18447581

[84] Naldini L, Blömer U, Gallay P, Ory D, Mulligan R, Gage FH, Verma IM, Trono D (1996). In vivo gene delivery and stable transduction of nondividing cells by a lentiviral vector. Science.

[85] Ghazawi A, Mustafa F, Phillip PS, Jayanth P, Ali J, Rizvi TA (2006). Both the 5’ and 3’ LTRs of FIV contain minor RNA encapsidation determinants compared to the two core packaging determinants within the 5’ untranslated region and gag. Microbes Infect.

