# Supplementary material for: Structural basis of genomic RNA (gRNA) dimerization and packaging determinants of mouse mammary tumor virus (MMTV)
Source: Retrovirology. 2014 Nov 14;11:96. doi: 10.1186/s12977-014-0096-6 (PMC4264320; doi:10.1186/s12977-014-0096-6)

Additional File 1

A

```
                                                  U5 LRI
AF228550.1    GCAACAGUCCUAAUAUUCACGUCUCGUGUGUUUGUGUCUGUUCGCCAUCCCGUCUCCGCU 60
L37517.1      ------GUCCUAACAUACACGUCUCGUGUGUUUGUGUCUGUUCGCCAUCCCGUCUCCGCU 54
AF033807.1    GCAACAGUCCUAACAUUCACCUCUCGUGUGUUUGUGUCUGUUCGCCAUCCCGUCUCCGCU 60
M15122.1      GCAACAGUCCUAACAUUCACCUCUCGUGUGUUUGUGUCUGUUCGCCAUCCCGUCUCCGCU 60
D16249.1      GCAACAGUCCUAACAUCCGUCUCUCGUGUGUUUGUGUCUGUUCGCCAUCCCGUCUCCGCU 60
AF228551.1    GCAACAGUCCUAAUAUUCACGUCUCGUGUGUUUGUGUCUGUUCGCCAUCCCGUCUCCGCU 60
X00018.1      GCAACAGUCCUAACAUUCUUCUCUCGUGUGUUUGUGUCUGUUCGCCAUCCCGUCUCCGCU 60
AF228552.1    GCAACAGUCCUAACAUUCACCUCUUGUGUGUUUGUGUCUGUUCGCCAUCCCGUCUCCGCU 60
                    ******* ** *   *** ***********************************
                                                                    pal I
AF228550.1    CGUCACUUAUCCUUCACUUUCCAGAGGGUCCCCCCGCAGACCCCGGUGACCCUCAGGUCG 120
L37517.1      CGUCACUUAUCCUUCACUUUCCAGAGGGUCCCCCCGCAGACCCCGGUGACCCUCAGGUCG 114
AF033807.1    CGUCACUUAUCCUUCACUUUCCAGAGGGUCCCCCCGCAGACCCCGGUGACCCUCAGGUCG 120
M15122.1      CGUCACUUAUCCUUCACUUUCCAGAGGGUCCCCCCGCAGACCCCGGUGACCCUCAGGUCG 120
D16249.1      CGUCACUUAUCCUUCACUUUCCAGAGGGUCCCCCCGCAGACCCCGGUGGCCCUCGGGUUC 120
AF228551.1    CGUCACUUAUCCUUCACUUUCCAGAGGGUCCCCCCGCAGACCCCGGUGACCCUCAGGUCG 120
X00018.1      CGUCACUUAUCCUUCACUUUUCAGAGGGUCCCCCCGCAGAUCCCGGUCACCCUCAGGUCG 120
AF228552.1    CGUCACUUAUCCUUCACUUUCCAGAGGGUCCCCCCGCAGACCCCGGCGACCCUCAGGUCG 120
              ******************** ******************* *****  .*****.***
                   pal sequences within PBS
AF228550.1    GCCGACUGCGGCAGCUGGCGCCCGAACAGGGACCCUCGGAUAAGUGACCCUUGUCUCUAU 180
L37517.1      GCCGACUGCGGCAGCUGGCGCCCGAACAGGGACCCUCGGAUAAGUGACCCUUGUCUCUAU 174
AF033807.1    GCCGACUGCGGCAGCUGGCGCCCGAACAGGGACCCUCGGAUAAGUGACCCUUGUCUCUAU 180
M15122.1      GCCGACUGCGGCAGCUGGCGCCCGAACAGGGACCCUCGGAUAAGUGACCCUUGUCUCUAU 180
D16249.1      GCCGACUGCGGCAGCUGGCGCCCGAACAGGGACCCUCGGAUAAGUGACCCUUGUCUCUAU 180
AF228551.1    GCCGACUGCGGCAGCUGGCGCCCGAACAGGGACCCUCGGAUAAGUGACCCUUGUCUUUAU 180
X00018.1      GCCGACUGCGGCAGCUGGCGCCCGAACAGGGACCCUCGGAUAAGUGACCCUUGUCUUUAU 180
AF228552.1    GCCGACUGCGGCAGCUGGCGCCCGAACAGGGACCCUCGGAUAAGUGACCCUUGUCUCUAU 180
              ******************************************************** ***
                                    PBS
AF228550.1    UUCUACUAUUUGGUGUUUGUCUUGUAUUGUCUCUUUCUUGUCUGGCUAUCAUCACAAGAG 240
L37517.1      UUCUACUAUUUGGUGUUCGUCUUGUAUUGUCUCUUUCUUGUCUGGCUAUCAUCACAAGAG 234
AF033807.1    UUCUACUAUUUUGUGUUCGUCUUGUUUUGUCUCUAUCUUAUCUGGCUAUUAUCACAAGAG 240
M15122.1      UUCUACUAUUUUGUGUUCGUCUUGUUUUGUCUCUAUCUUAUCUGGCUAUUAUCACAAGAG 240
D16249.1      UUCUACUAUUUGGUGUUCGUCUUGUAUUGUCUCUUUCUUGUCUGGCUAUCAUCACAAGAG 240
AF228551.1    UUCUACUAUUUUGUGUUCGUCUUGUUUUGUCUCUAUCUUGUCUGGCUAUCAUCACAAGAG 240
X00018.1      UUCUACUAUUUUGUGUUCGUCUUGUUUUGUCUCUAUCUUGUCUGGCUAUCAUCACAAGAG 240
AF228552.1    UUCUACUAUUUGGUGUUUGUCUUGUAUUGUCUCUUUCUUGUCUGGCUAUCAUCACAAGAG 240
              *********** ***** *******:********:****.********* **********
                                    pal II             ssPurines
AF228550.1    CGGAACGGACUCACCAUAGGGAGCUGCAGUCCCGCCUACGGAGAAGAGGUAGGUUACGGU 300
L37517.1      CGGAACGGACUCACCACAGGGAGCUGCAGUCCCGCCUACGGAGGAGAGGUAGGUUACGGU 294
AF033807.1    CGGAACGGACUCACCACAGGGAACUGCAGUCUCGCCUACAGAGAAGAGGUAGGUUACGGU 300
M15122.1      CGGAACGGACUCACCACAGGGAACUGCAGUCUCGCCUACAGAGAAGAGGUAGGUUACGGU 300
D16249.1      CGGAACGGACUCACCACAGGGAACUGCAGUCCCGCCUACGGAGGAGAGGUAGGUUACGGU 300
AF228551.1    CGGAACGGACUCACCACAGGGAACUGCAGUCUCGCCUACGGAGAAGAGGUAGGUUACGGU 300
X00018.1      CGGAACGGACUCACCACAGGGAACUGCAGUCUCGCCUACGGAGAAGAGGUAGGUUACGGU 300
AF228552.1    CGGAACGGACUCACCAUAGGGAGCUGCAGUCCCGCCUACGGAGAAGAGGUAGGUUACGGU 300
              **************** *****.******** *******.***.****************

                         Gag LRI
AF228550.1    GAGCCAUUGGAAAUGGGGGUCUCGGGCUCAAAAGGGCAGAAACUCUUUGUUUCUGUUUUA 360
L37517.1      GAGCCAUUGGAUAUGGGGGUCUCGGGCUCAAAAGGGCAGAAACUCUUCGUUUCUGUACUA 354
AF033807.1    GAGCCAUUGGAAAUGGGGGUCUCGGGAUCAAAAGGGCAGAAACUCUUUGUUUCUGUUCUA 360
M15122.1      GAGCCAUUGGAAAUGGGGGUCUCGGGAUCAAAAGGGCAGAAACUCUUUGUUUCUGUUCUA 360
D16249.1      GAGCCAUUGGAAAUGGGGGUCUCGGGCUCAAAAGGGCAGAAACUCUUCGUUUCUGUUUUA 360
AF228551.1    GAGCCAUUGGAAAUGGGGGUCUCGGGCUCAAAAGGGCAGAAACUCUUUGUUUCUGUUUUA 360
X00018.1      GUGCCAUUGGAUAUGGGGGUCUCGGGCUCAAAAGGGCAGAAACUCUUUGUUUCUGUUUUA 360
AF228552.1    GAGCCAUUGGAAAUGGGGGUCUCGGGCUCAAAAGGGCAGAAACUCUUUGUUUCUGUUUUA 360
              *:*********:**************.******************** ********: **
                      pal III
AF228550.1    CAAAGGCUCCUCUCAGAGAGGGGUCUUCAUGUGAAAGAGAGUAGUGCAAUAGAAUUUUAU 420
L37517.1      CAAAGGCUCCUCUCAGAGAGGGGUCUUCAUGUGAAAGAGAGUAGUGCAAUAGAAUUUUAU 414
AF033807.1    CAAAGACUCCUCUCAGAGAGGGGUCUUCAUGUGAAAGAGAGCAGUGCAAUAGAGUUUUAU 420
M15122.1      CAAAGACUCCUCUCAGAGAGGGGUCUUCAUGUGAAAGAGAGCAGUGCAAUAGAGUUUUAU 420
D16249.1      CAAAGGCUUCUCUCAGAGAGGGGUCUUCAUGUGAAAGAGAGCAGUGCAAUAGAAUUCUAU 420
AF228551.1    CAAAGGCUCCUCUCAGAGAGGGGUCUUCAUGUGAAAGAGAGCAGCGCAAUAGAGUUUUAU 420
X00018.1      CAAAGGCUCCUCUCAGAGAGGGGUCUUCAUGUGAAGGAGAGCAGCACAAUAGAGUUUUAU 420
AF228552.1    CAAAGGCUCCUCUCAGAGAGGGGUCUUCAUGUGAAAGAGAGUAGUGCAAUAGAAUUUUAU 420
              *****.** **************************.***** ** .*******.** ***

AF228550.1    CAGUUUCUAAUA 432
L37517.1      CAGUUUCUGAUA 426
AF033807.1    CAGUUCCUAAUA 432
M15122.1      CAGUUCCUAAUA 432
D16249.1      CAGUUCCUAAUA 432
AF228551.1    CAGUUUCUUAUA 432
X00018.1      CAGUUUCUGAUA 432
AF228552.1    CAGUUUCUUAUA 432
              ***** ** ***
```

B

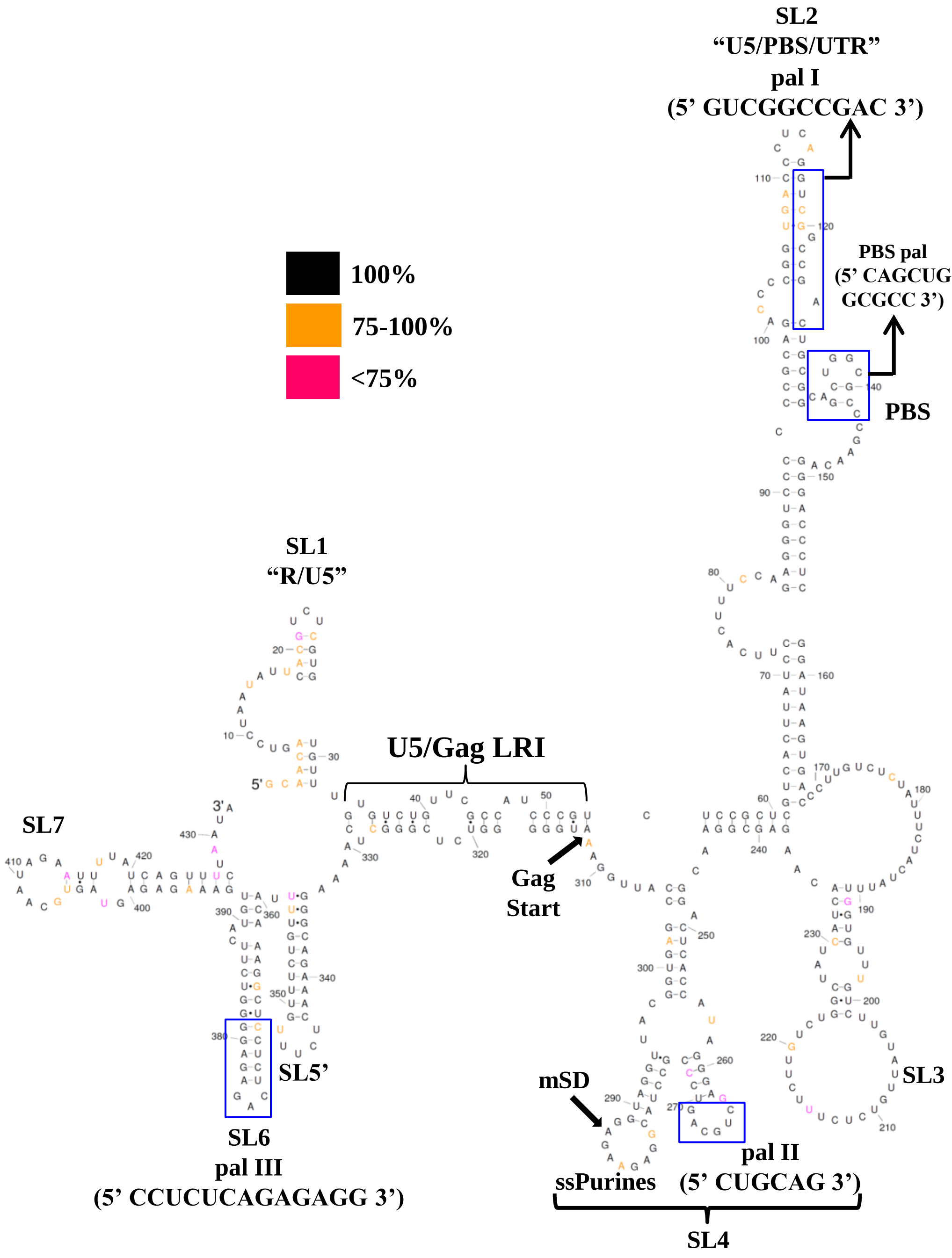

Supplement: Additional file 1 — Phylogenetic conservation of the sequences of major structural motifs of MMTV packaging signal RNA in different strains. (A) Clustal Omega sequence alignment of eight different strains of MMTV packaging signal RNA. Sequences of the major structural motifs are highlighted in different colors, whereas the 11 nt pal sequence within PBS is boxed. The accession numbers for the MMTV strains are provided in the Materials and Methods. (B) Sequence conservation superimposed on the SHAPE validated structure. The nucleotides are color-coded based on their conservation level, ranging from <75 to 100%. [file 12977_2014_96_MOESM1_ESM.pdf]
