# Supplementary figures and images for: Structural basis of genomic RNA (gRNA) dimerization and packaging determinants of mouse mammary tumor virus (MMTV)
Source: Retrovirology. 2014 Nov 14;11:96. doi: 10.1186/s12977-014-0096-6 (PMC4264320; doi:10.1186/s12977-014-0096-6)

# Additional File 2

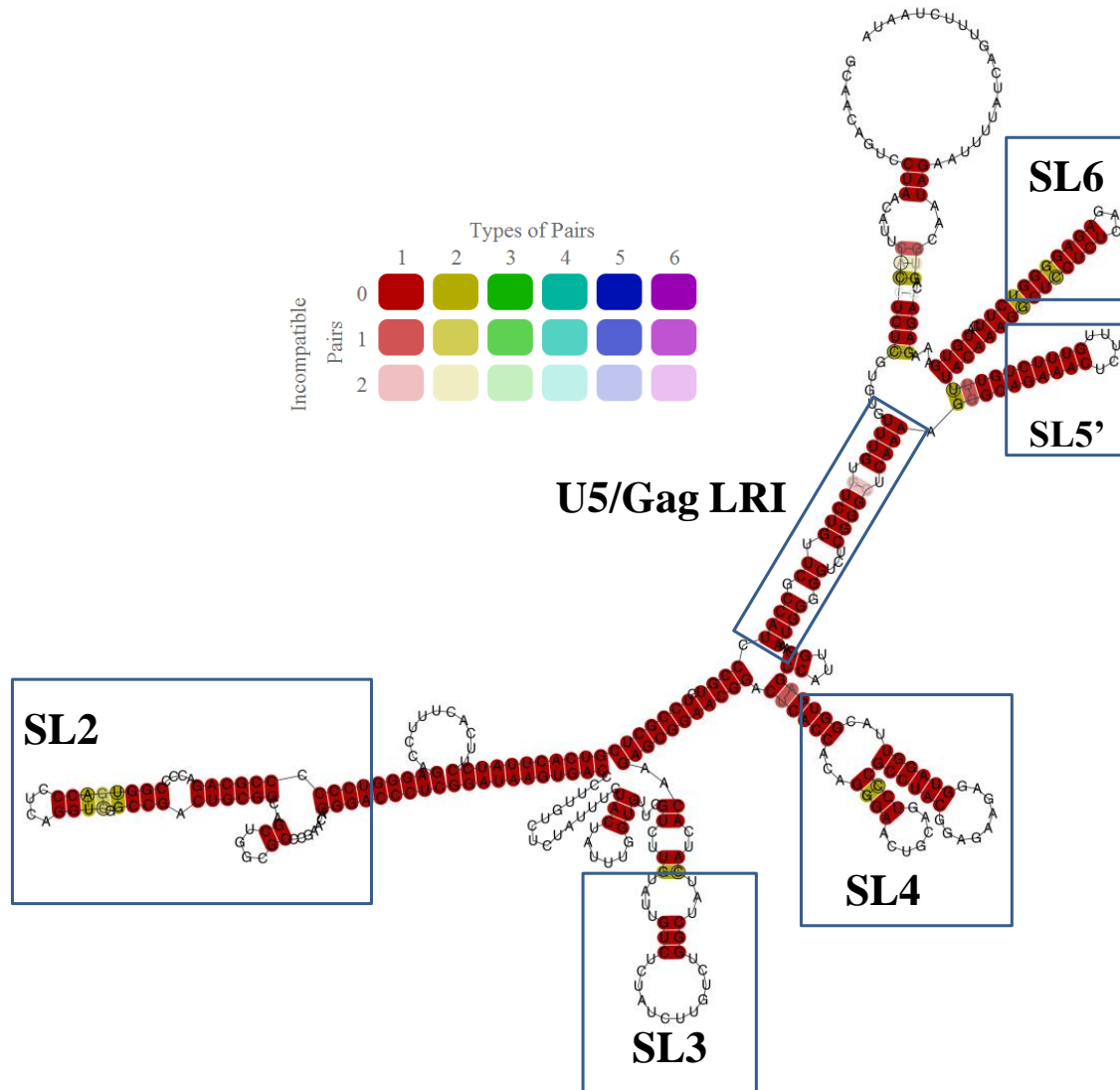

Supplement: Additional file 2 — Consensus RNA secondary structure prediction of MMTV packaging signal RNA using sequences from eight different strains. Accession numbers of MMTV strains are the same as mentioned in materials and methods section. The sequences of the 8 MMTV strains were aligned using Clustal Omega and the aligned sequences were used as input in the RNAalifold server [73,74] to predict the consensus or “conservation annotation” structure. The number/color scale on the x-axis indicates base pairing conservation with 1 (red color) representing completely conserved base pairing to 6 (violet color) representing 6 different types of base pairings at that position among the various strains (see Methods for details). The y-axis represents the incompatibility score based on non-Watson base pairing within each strain. The number (0-2) or color gradation (dark to very pale) represents increasing number of strains showing incompatible (non-Watson) base pairing within the structure of the various strains. Thus, a dark red color denotes “highest” conservation of base pairings, while a light violet color represents “least” conservation of base pairing among different strains of MMTVs. [file 12977_2014_96_MOESM2_ESM.pdf]

# Additional File 3

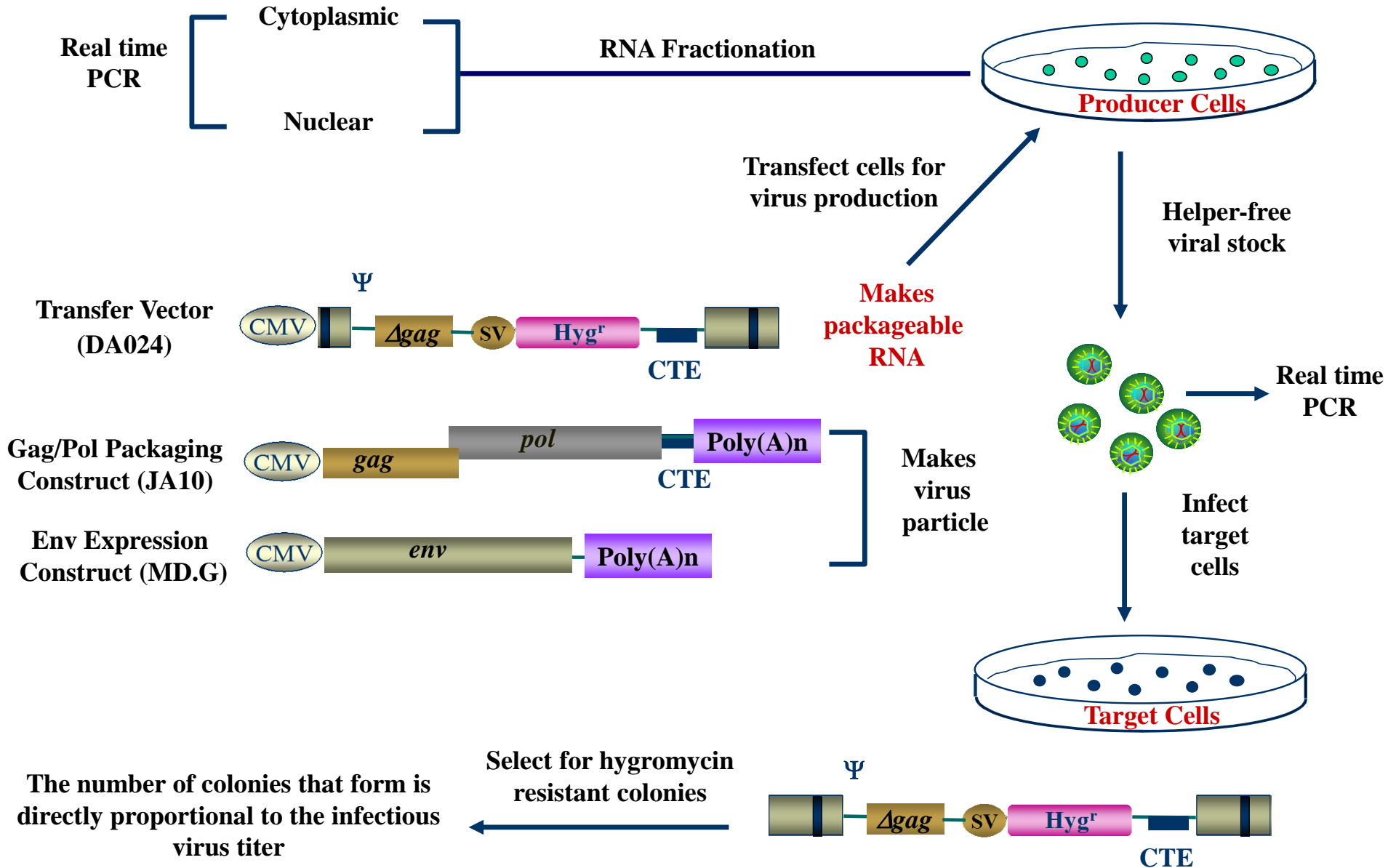

Supplement: Additional file 3 — Schematic representation of the design and rationale of three plasmid trans complementation assay for studying MMTV gRNA packaging and propagation developed earlier [ 45 ]. The pseudotyped particles produced following transfection of human embryonic kidney (HEK) 293T cells with MMTV Gag/Pol expression plasmid (JA10) and VSV-G envelope expression plasmid (MD.G) allows packaging of the transfer vector RNA by virtue of the presence of the packaging signal (ψ) on the MMTV transfer vector (DA024). Virus particles produced following transfection contain the packaged transfer vector RNA (DA024) and are used to quantitate RNA packaging by real time PCR or to infect target cells to monitor RNA propagation. [file 12977_2014_96_MOESM3_ESM.pdf]
