# Supplementary material for: Structural basis of genomic RNA (gRNA) dimerization and packaging determinants of mouse mammary tumor virus (MMTV)
Source: Retrovirology. 2014 Nov 14;11:96. doi: 10.1186/s12977-014-0096-6 (PMC4264320; doi:10.1186/s12977-014-0096-6)
Supplement: Additional file 4 — Table describing primers used for cloning, sequencing, and conventional and real time PCR. [file 12977_2014_96_MOESM4_ESM.pdf]

Additional File 4. Description of primers used for cloning, sequencing, and conventional and real time PCR

Oligo Name	S/ AS	Clone Name	Sequence	Nucleotide Position (nt)	Virus & Gene (or plasmid)
OTR 984	S	SA035	5’cccaagcttAATACGACTCACTATAGGGCAACAGTC CTAATATTCACG 3’	1173-1193	HYBMTV R (+1) start
OTR 985	AS		5’aaacccgggTTCCCCTGGTCCCATAAG 3’	1867-1885	HYBMTV *gag*
OTR 922	S	SA031/ SA031P	5’CCCCGCAGATGCGGCAGCTGGCGC 3’	1264-1313	HYBMTV U5 region
OTR 923	AS		5’ GCTGCCGCATCTGCGGGGGGACCC 3’	1258-1307	HYBMTV U5 region
OTR 924	S	SA032/ SA032P	5’ TTCGTGCGGCAGCTGGCGCCCG 3’	1299-1322	HYBMTV U5 region
OTR 925	AS		5’ GCCGCACGAATCTGCGGGGGGACCCTCTGGA 3’	1304-1252	HYBMTV U5 region
OTR 926	S	SA033/ SA033P	5’ CCCCCCGCAGCTGCGGCAGCTGGCGC 3’	1260-1313	HYBMTV U5 region
OTR 927	AS		5’ GCTGCCGCAGCTGCGGGGGGACCCTCTG 3’	1307-1254	HYBMTV U5 region
OTR 986	S	SA041/ SA041P	5' GTAATCCCGCCTACGGAGAAGAGGTAG 3'	1442-1464	HYBMTV UTR region
OTR 987	AS		5' GCGGGATTACTCCCTATGGTGAGTCCGTTCC 3'	1434-1414	HYBMTV UTR region
OTR 988	S	SA042/ SA042P	5' CACCATAGCCTACGGAGAAGAGGTAGG 3'	1446-1465	HYBMTV UTR region
OTR 989	AS		5' GTAGGCTATGGTGAGTCCGTTCCGCTC 3'	1430-1410	HYBMTV UTR region
OTR 992	S	SA044/ SA044P	5' GACGCACTCCCGCCTACGGAGAAGAGG 3'	1442-1461	HYBMTV UTR region
OTR 993	AS		5' GCGGGAGTGCGTCTCCCTATGGTGAGTCCGTTCC 3'	1434-1414	HYBMTV UTR region
OTR 994	S	SA045/ SA045P	5' GGTGCGTTCCCGCCTACGGAGAAGAGG 3'	1442-1461	HYBMTV UTR region
OTR 995	AS		5' GCGGGAACGCACCTCCCTATGGTGAGTCCGTTCC 3'	1434-1414	HYBMTV UTR region
OTR 1075	S	SA046/ SA046P	5’ CGACTGCGGCGAACAGGGACCCTCGG 3’	1315-1331	HYBMTV UTR region
OTR 1076	AS		5’ CCTGTTCGCCGCAGTCGGCCGACC 3’	1303-1288	HYBMTV UTR region
OTR 1077	S	SA047/ SA047P	5’ AAGCGCGCATCCCGCCTACGGAGAAGAGG 3’	1442-1461	HYBMTV UTR region
OTR 1078	AS		5’ GCGGGATGCGCGCTTCTCCCTATGGTGAGTCCGTTC 3’	1435-1415	HYBMTV UTR region
OTR 1200	S	SA052	5'AAACGCACATCCCGCCTACGGAGAAGAGG 3'	1442-1461	HYBMTV UTR region
OTR 1201	AS		5'GCGGGATGTGCGTTTCTCCCTATGGTGAGTCCG TTCC 3'	1433-1414	HYBMTV UTR region
OTR 1202	S	SA053	5'AAGTGCGTATCCCGCCTACGGAGAAGAGG 3'	1442-1461	HYBMTV UTR region
OTR 1203	AS		5'GCGGGATACGCACTTCTCCCTATGGTGAGTCCGTT CC 3'	1433-1414	HYBMTV UTR region
OTR 1208	S	SA056	5'AAGCGCGCACGAACAGGGACCCTCGG 3'	1315-1331	HYBMTV UTR region
OTR 1209	AS		5'CCTGTTCGTGCGCGCTTCCGCAGTCGGCCGACC 3'	1303-1288	HYBMTV UTR region

OTR 1204	S	SA057	5'AAACGCACACGAACAGGGACCCTCGG 3'	1315-1331	HYBMTV UTR region
OTR 1205	AS		5'CCTGTTCGTGTGCGTTTCCGCAGTCGGCCGACC 3'	1303-1288	HYBMTV UTR region
OTR 1206	S	SA058	5'AAGTGCGTACGAACAGGGACCCTCGG 3'	1315-1331	HYBMTV UTR region
OTR 1207	AS		5'CCTGTTCGTACGCACTTCCGCAGTCGGCCGACC 3'	1303-1288	HYBMTV UTR region
OTR 1224	S	SA059	5'AAGCGCCCACGAACAGGGACCCTCGG 3'	1315-1331	HYBMTV UTR region
OTR 1225	AS		5'CCTGTTCGTGGGCGCTTCCGCAGTCGGCCGACC 3'	1303-1288	HYBMTV UTR region
OTR 1226	S	SA060	5'AAGGGCGCACGAACAGGGACCCTCGG 3'	1315-1331	HYBMTV UTR region
OTR 1227	AS		5'CCTGTTCGTGCGCCCTTCCGCAGTCGGCCGACC 3'	1303-1288	HYBMTV UTR region
OTR 1212	S	SA061	5' GGTCTTCATGTGAAAGAGAG 3'	1554-1573	HYBMTV *gag*
OTR 1213	AS		5' CTCTTTCACATGAAGACCAGCCTTTGTAAAACAGAAACAAAG 3'	1540-1517	HYBMTV *gag*
OTR 930	S	Used for sequencing	5’ GCTTGTGTGTTGGAGGTCGCTGAG 3'	90-113	pcDNA3
OTR 725	S		5’ aaaaaagcggccgcCCCTCGGATAAGTGACCCTTGTC 3’	1325-1347	HYBMTV UTR region
OTR 581	AS	β-actin	5’ GGCATGGGGGAGGGCATACC 3’		Actin A. Spliced or unspliced mRNA
OTR 582	S	β-actin	5’ CCAGTGGCTTCCCCAGTG 3’		Actin S-1. Unspliced mRNA
OTR 671	S	For PCR following DNase treatment	5’ GTCCTAATATTCACGTCTCGTGTG 3’	1179-1202	HYBMTV R region
OTR 672	AS	For PCR following DNase treatment	5’ CTG TTCGGGCGCCAGCTGCCGCAG 3’	1321-1298	HYBMTV PBS region
MTV-1LTR-SITEM1 FAM	-	qPCR probe	5’TCGCCATCCCGTCTCC 3’	1214-1229	HYBMTV U5 region
MTV-1LTR-SITEF	S	qPCR Forward primer	5’CGTCTCGTGTGTTTGTGTTTGTGTCTGT 3’	1192-1213	HYBMTV U5 region
MTV-1LTR-SITER	AS	qPCR Reverse Primer	5’CCTCTGGAAAGTGAAGGATAAGTGA 3’	1259-1235	HYBMTV U5 region

Underlined sequence: T7 promoter.
Sequence in lower case: artificial and restriction enzyme sequences that were introduced in the oligos.
*S, sense; AS, antisense.
**HYBMTV, MMTV molecular clone created by Shackleford and Varmus [70].
