# Supplementary material for: Structural basis of genomic RNA (gRNA) dimerization and packaging determinants of mouse mammary tumor virus (MMTV)
Source: Retrovirology. 2014 Nov 14;11:96. doi: 10.1186/s12977-014-0096-6 (PMC4264320; doi:10.1186/s12977-014-0096-6)
Supplement: Additional file 5 — Normalized SHAPE reactivity data from at least three to six independent experiments. [file 12977_2014_96_MOESM5_ESM.pdf]

Additional File 5: Normalized SHAPE reactivity data from at least 3-6 independent experiments.

Nt no.	RNA seq	BzCN reactivity
1	G	-999
2	C	-999
3	A	-999
4	A	-999
5	C	-999
6	A	-999
7	G	-999
8	U	-999
9	C	-999
10	C	-999
11	U	0.284965
12	A	0.30384
13	A	0.374618
14	U	0.598795
15	A	1.106763
16	U	0.860522
17	U	0.468574
18	C	0
19	A	0.080154
20	C	0.043616
21	G	0.541723
22	U	1.596105
23	C	0.523824
24	U	1.915072
25	C	0.537045
26	G	0.183978
27	U	0.041542
28	G	0.025346
29	U	0.042334
30	G	0.131507
31	U	0.053142
32	U	0.192344
33	U	0.132093
34	G	0.338396
35	U	1.088123
36	G	0.231608

37	U	0.044274
38	C	0.046582
39	U	0.033023
40	G	0.045187
41	U	0.068223
42	U	0.339979
43	C	0.363275
44	G	0.04309
45	C	0.08158
46	C	0.269493
47	A	0.947545
48	U	0.48476
49	C	0.146278
50	C	0.100549
51	C	0.111207
52	G	0.18634
53	U	0.25804
54	C	0.373532
55	U	0.111575
56	C	0.023287
57	C	0.02147
58	G	0.049451
59	C	0.045637
60	U	0.180133
61	C	0.302482
62	G	0.168699
63	U	0.141848
64	C	0.164461
65	A	0.165159
66	C	0.003774
67	U	0.170392
68	U	0.302176
69	A	0.264256
70	U	0.145866
71	C	0.073816
72	C	0.188853
73	U	0.74335
74	U	0.765415
75	C	0.458458
76	A	0.68438
77	C	0.318862
78	U	0.547803
79	U	0.597699

80	U	0.195019
81	C	0.015805
82	C	0.115825
83	A	0.279112
84	G	1.135396
85	A	0.135927
86	G	0.087179
87	G	0.03681
88	G	0.095094
89	U	0.05123
90	C	0.01639
91	C	0.070724
92	C	0.184247
93	C	0.055354
94	C	0.088033
95	C	0.142484
96	G	0.124226
97	C	0.238904
98	A	0.35033
99	G	0.62501
100	A	0.755467
101	C	0.096648
102	C	0.140551
103	C	-999
104	C	0.133437
105	G	0.040354
106	G	0.136073
107	U	0.186143
108	G	0.046355
109	A	0.05508
110	C	0.034967
111	C	0.214967
112	C	0.506359
113	U	1.14652
114	C	0.879529
115	A	1.907796
116	G	0.165538
117	G	0.096755
118	U	0.132462
119	C	0.044327
120	G	0.59241
121	G	0.347825
122	C	0.031135

123	C	0.033952
124	G	0.340301
125	A	0.208174
126	C	0.077362
127	U	0.124261
128	G	0.124444
129	C	0.081912
130	G	0.073219
131	G	0.069411
132	C	0.048728
133	A	0.259639
134	G	0.394971
135	C	0.981856
136	U	0.446584
137	G	0.182419
138	G	0.418695
139	C	0.239696
140	G	0.175224
141	C	0.027418
142	C	0.114856
143	C	0.422388
144	G	0.559742
145	A	1.097897
146	A	0.870001
147	C	0.700993
148	A	0.551254
149	G	0.053104
150	G	0.05612
151	G	0.083136
152	A	0.17364
153	C	-999
154	C	-999
155	C	-999
156	U	0.235599
157	C	0.233505
158	G	0.065587
159	G	0.057254
160	A	0.276932
161	U	0.457166
162	A	0.06286
163	A	0.032862
164	G	0.043947
165	U	0.17765

166	G	0.095339
167	A	0.204016
168	C	0.21478
169	C	0.131542
170	C	0.166307
171	U	0.336647
172	U	0.32875
173	G	0.71542
174	U	0.480194
175	C	0.087099
176	U	0.121883
177	C	0.128615
178	U	0.421845
179	A	0.236454
180	U	0.232347
181	U	0.33228
182	U	0.201789
183	C	0.254199
184	U	0.421595
185	A	0.278185
186	C	0.21766
187	U	0.560351
188	A	0.532074
189	U	0.22251
190	U	0.207384
191	U	0.314571
192	G	0.095631
193	G	0.330806
194	U	0.366171
195	G	0.495795
196	U	0.382108
197	U	0.478709
198	U	0.333684
199	G	0.276521
200	U	0.170637
201	C	0.164966
202	U	0.399223
203	U	0.321233
204	G	0.285345
205	U	0.367735
206	A	0.248367
207	U	0.355475
208	U	0.452699

209	G	0.686958
210	U	0.1688
211	C	0.150061
212	U	0.072372
213	C	0.069265
214	U	0.292765
215	U	0.318383
216	U	0.245206
217	C	0.093581
218	U	0.504522
219	U	0.296694
220	G	0.345326
221	U	0.228213
222	C	0.148485
223	U	0.204951
224	G	0.183051
225	G	0.03186
226	C	0.106517
227	U	0.446862
228	A	0.257258
229	U	0.164891
230	C	0.520905
231	A	0.55358
232	U	0.239523
233	C	0.550044
234	A	0.607877
235	C	0.840358
236	A	0.775564
237	A	0.639879
238	G	0.296998
239	A	0.234025
240	G	0.113424
241	C	0.139225
242	G	0.23025
243	G	0.202711
244	A	0.322454
245	A	0.289909
246	C	0.062584
247	G	-999
248	G	-999
249	A	0.806104
250	C	0.190316
251	U	0.217485

252	C	0.111816
253	A	0.112216
254	C	0.061862
255	C	0.184081
256	A	1.124839
257	U	0.722007
258	A	0.533839
259	G	0.250752
260	G	0.120762
261	G	0.218423
262	A	0.195615
263	G	0.205473
264	C	0.174125
265	U	0.387671
266	G	0.290372
267	C	0.78527
268	A	0.754735
269	G	0.353313
270	U	0.289098
271	C	0.160926
272	C	0.148276
273	C	0.084039
274	G	0.110905
275	C	0.307071
276	C	0.334201
277	U	0.295669
278	A	0.06084
279	C	0.108063
280	G	0.220021
281	G	0.780514
282	A	1.247749
283	G	0.845051
284	A	1.354653
285	A	1.382226
286	G	1.090855
287	A	0.881956
288	G	0.747197
289	G	0.139271
290	U	0.245262
291	A	0.031245
292	G	0.082617
293	G	0.240763
294	U	0.779868

295	U	0.597589
296	A	0.505625
297	C	0.239014
298	G	0.178263
299	G	0.06438
300	U	0.204746
301	G	0.12063
302	A	0.065894
303	G	0.075554
304	C	0.174238
305	C	0.563284
306	A	0.676458
307	U	0.552505
308	U	0.680604
309	G	0.447293
310	G	0.50253
311	A	0.427984
312	A	0.352168
313	A	0.39676
314	U	0.234197
315	G	0.086429
316	G	0
317	G	0.058677
318	G	0.147937
319	G	0.323339
320	U	0.309459
321	C	0.157266
322	U	0.138762
323	C	0.24083
324	G	0.306462
325	G	0.299193
326	G	0.193736
327	C	0.088351
328	U	0.446606
329	C	1.010401
330	A	0.484196
331	A	0.765479
332	A	0.678972
333	A	0.509723
334	G	0.185994
335	G	0.102755
336	G	0.139023
337	C	0.060567

338	A	0.115974
339	G	0.160287
340	A	0.213725
341	A	0.288594
342	A	0.177028
343	C	0.134095
344	U	0.457414
345	C	0.404796
346	U	0.58935
347	U	0.692395
348	U	0.630118
349	G	0.026645
350	U	0.141345
351	U	0.040344
352	U	0
353	C	0.025843
354	U	0.490221
355	G	0.700606
356	U	0.385538
357	U	0.350645
358	U	0.333142
359	U	0.320929
360	A	0.516689
361	C	0.87976
362	A	0.228858
363	A	0.193516
364	A	0.145364
365	G	0.033957
366	G	0.117133
367	C	0.084613
368	U	0.038526
369	C	0.026351
370	C	0.058223
371	U	0.080661
372	C	0.094581
373	U	0.362298
374	C	1.259264
375	A	1.737467
376	G	1.342181
377	A	1.306808
378	G	0.043065
379	A	0.023706
380	G	0

381	G	-999
382	G	0
383	G	-999
384	U	0
385	C	-999
386	U	0.234021
387	U	0.745919
388	C	1.383041
389	A	0.813116
390	U	0.198127
391	G	0.082211
392	U	0.074206
393	G	0.076748
394	A	0.125872
395	A	0.25386
396	A	0.377037
397	G	0.275605
398	A	0.124367
399	G	0.134431
400	A	0.086701
401	G	0.109682
402	U	0.486064
403	A	0.197638
404	G	0.150322
405	U	0.274143
406	G	0.718776
407	C	1.4075
408	A	0.727748
409	A	0.510831
410	U	0.626919
411	A	0.47635
412	G	0.477619
413	A	0.500571
414	A	0.373856
415	U	0.359908
416	U	0.259863
417	U	0.501968
418	U	0.389608
419	A	0.30641
420	U	0.152996
421	C	0.981109
422	A	0.608906
423	G	0.403632

424	U	0.247558
425	U	0.340032
426	U	0.137758
427	C	0.158769
428	U	0.794397
429	A	0.181424
430	A	0.551719
431	U	0.795339
432	A	0.731406
